# Supplementary material for: Evaluation of a new transpalpebral tonometer for self-measuring intraocular pressure
Source: PLoS One. 2024 May 15;19(5):e0302568. doi: 10.1371/journal.pone.0302568 (PMC11095731; doi:10.1371/journal.pone.0302568)
Supplement: S1 File — (PDF) [file pone.0302568.s002.pdf]

正常者及び緑内障患者に対する新しい自己測定眼圧計

タップアイの眼圧変化の探索的試験

研究計画書

実施医療機関：医療法人仁慈会 横浜鶴見中央眼科
研究責任医師：勅使川原 剛
医療機関住所：〒230-0051 神奈川県横浜市鶴見区鶴見中央 1 丁目 2-1

研究実施予定期間：実施計画の公表日～2023 年 12 月 31 日

版数　：第 1.0 版
作成日：2022 年 7 月 1 日

略語及び用語の定義一覧

略語	略さない表現
IOP	Intraocular pressure（眼圧）
NCT	Non-contact tonometer（非接触型眼圧計）
GAT	Goldmann applanation tonometer（ゴールドマン眼圧計）
TET	Tapeye tonometer　（タップアイ眼圧計）

用語	定義

目次

要約..4
1 実施体制..5
2 背景情報..6
3 目的..133
4 研究デザイン..133
5 研究対象者の選択・除外・中止基準..16
6 研究の方法..17
7 有効性の評価..17
8 安全性の評価..18
9 統計解析..21
10 モニタリング..23
11 倫理的な配慮..23
12 データの取り扱いと記録保存..25
13 研究対象者の経済的負担及び保険その他の措置..26
14 臨床研究に関する情報の公表に関する取決め..26
15 補遺..26

要約

0.1 シェーマ

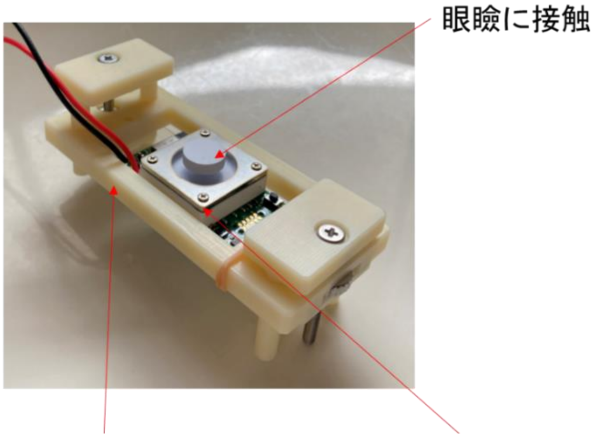

0.2 目的

正常眼及び緑内障眼を対象として自己測定眼圧計タップアイの測定精度・再現性を検討する。

0.3 対象

正常眼及び緑内障眼を対象とする。

0.4 試験治療

治療介入なし。

0.5 予定登録数と研究期間

合計 100 例、実施計画の公表日～2023 年 12 月 31 日

0.6 問い合わせ先

〒230-0051 神奈川県横浜市鶴見区鶴見中央 1 丁目 2-1
医療法人仁慈会 横浜鶴見中央眼科
北村　彰
Email：tsurumi.chuoh.eye.cs@gmail.com

1 実施体制

1.1 研究情報
正常者及び緑内障患者に対する新しい自己測定眼圧計タップアイの眼圧変化の探索的試験

認定臨床研究審査委員会：医療法人社団信濃会 信濃坂クリニック 臨床研究審査委員会

1.2 研究責任医師
氏名：勅使川原 剛
所属機関：医療法人仁慈会 横浜鶴見中央眼科
所属部署：医局
職位：医師
住所： 〒230-0051 神奈川県横浜市鶴見区鶴見中央 1 丁目 2-1
電話番号：045-508-1017
Email：teshitake@gmail.com

1.3 データマネジメントに関する責任者
氏名：川嵜莉奈
所属機関：長崎大学病院
所属部署：臨床研究センター
職位：データーマネジャー
住所：〒852-8501 長崎県長崎市坂本 1 丁目 7-1
電話番号：095-819-7726
Email：kawasaki@nagasaki-u.ac.jp

1.4 統計解析に関する責任者
氏名：水上 貴裕
所属機関：多根記念眼科病院（2023 年 4 月着任予定）
所属部署：眼科
職位：医師
住所：〒550-0024 大阪府大阪市西区境川１丁目１－３９
電話番号：06-6581-5800

1.5 モニタリングに関する責任者
氏名：富士井淳子
所属機関：株式会社新薬リサーチセンター
所属部署：研究本部 臨床研究部
職位：部長
住所：〒100-0006 東京都千代田区有楽町一丁目 7 番 1 号 有楽町電気ビル 南館 4 階
電話番号：03-6551-2335
Email：j-fujii@ndrcenter.co.jp

1.15 研究・開発計画支援担当機関
氏名：那谷由美
所属機関：株式会社 UMIN
所属部署：代表取締役
住所：〒194-0004 東京都町田市鶴間 1 丁目 2-6
電話番号：042-795-4812
Email：y-nata@umin.co.jp

2 背景情報

2.1 対象疾患の状況
　緑内障とは、視神経と視野に特徴的変化を有し、通常、眼圧を十分に降下させることにより視神経障害を改善もしくは抑制しうる眼の機能的、構造的異常を特徴とする疾患と定義される（日本緑内障ガイドライン）。緑内障は我が国における失明原因の上位を占める。2000 年～2002 年に行われた詳細な緑内障疫学調査(Iwase et al. Tajimi Study. Ophthalmology. 2004)では、40 歳以上の日本人における緑内障の有病率は 5.0％であり、2016 年の推定患者数は 465 万人に上る。緑内障では、患者の自覚なしに障害が徐々に進行するため、その早期発見と早期治療による障害の進行の阻止あるいは抑制が重要である。眼圧を降下させることが唯一の緑内障の治療とされており、点眼薬が使用されるが、眼圧には日内変動があり適切なタイミングでの点眼が難しい。常に眼圧を把握したいが、患者は頻繁に医療機関にアクセスできず、眼圧を常に把握できるわけではない。もし患者が自宅でも眼圧計測を簡便に安全にできれば、(1)治療効果が評価し易くなり服薬アドヒアランスが向上する。(2)緑内障の更なる病態解明に寄与する。(3)眼圧を降下させる点眼薬を必要な時（眼圧上昇時）にだけ使用すれば医療費の削減につながる。

2.2 既存の眼圧計

現在本邦で認証されている眼圧計の一覧を下記に示す。

眼瞼を通して自己測定

	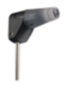	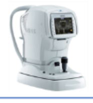	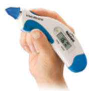	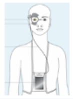	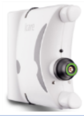	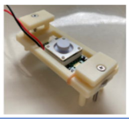
	Goldmann	ノンコン	トノペン	Triggerfish	iCareHome	タップアイ
接触	角膜	非接触	角膜	角膜	角膜	眼瞼
携帯性	×	×	○	○	○	◎
麻酔	要	不要	要	不要	不要	不要
侵襲	有り	少ない	有り	有り	少ない	無
精度	高い	やや低い	低い	やや低い	やや高い	やや高い
売価(円)	15万	100万	50万	5万円/CL1枚	100万円	未定
企業名	HAAG-STREIT (独)	Nidek (日)	Reichert (米)	SENSIMED (スイス)	icare (フィンランド)	弊社
特徴	ゴールドスタンダード	スクリーニング	携帯可能	付属品多い	自宅で可能	瞼を介して低侵襲 低価格
分類	クラスⅡ	クラスⅡ	クラスⅡ	クラスⅢ	クラスⅡ	新名称?
自己計測	×	×	×	△	○	◎
遠隔医療	×	×	×	×	×	○

2.3 臨床研究の必要性につながる、現在の課題、不明点等

エンドユーザーが自己測定できる上市されている眼圧計は iCare Home のみである。表に示すように iCare Home の短所は、１）自己測定するのにコツが必要であること、２）１台 100 万円と高額であること、３）角膜に直接プローブが当たるため、患者の恐怖心をあおることなどである。これらの課題を本機器タップアイは解決できる。

緑内障は早期発見・早期治療介入が失明を回避する上で重要であるため、本機器を使用することで眼圧データのモニタリングが容易になる。また、患者一人一台所有できる価格設定であるため点眼のアドヒアランスを向上させ、点眼後の効果も実感できる。最終的に緑内障治療にかかる医療費の増大抑制に貢献できる。

2.4 臨床研究に用いる当該研究機器等に関する以下の情報

2.4.1 研究機器

自己測定眼圧計タップアイ

MEMS（Micro Electro Mechanical Systems、微小電子機械システム）とはシリコンウェハーなどの上に、電子回路やセンサ、機械的に動くアクチュエーターなどを作りこんだ部品である。MEMS を搭載した代表的な利用例は自動車やスマートフォンである。本機器は、瞼を介した押圧による変化量・反発力を複数 MEMS センサと専用ケースを用いて計測して眼圧値として表示する眼圧計である。現在、MEMS センサを用いた眼圧計は市販されていない。既存の眼圧計の動作原理に共通することは、測定対象物の変形量に対する反発力を

測定する点である。本機器は**図 2-1** のように、変形量を知るための加速度センサ(加速度の二重積分は距離)、反発力を知るための気圧センサと一面だけがたわむ密封空間(内圧と反発力は一対一の対応)、回転による重力の向きを補正するジャイロセンサ（加速度センサと同一の IC パッケージ)、外部気圧が変わってもゆっくりと通気させ内外気圧差を無視できるようにするためのベントフィルタから成る。変形量と反発力との比に補正式を適用したものを眼圧とみなす。下面に緩衝材を配置し、軽くたたくことにより、本体をまぶたの方向に押圧する。不要な回転にともなう重力の向きの変化は、ジャイロセンサにより補正する。適切な通気量のベントフィルタ(空気は通すが水は通さない膜)により、空気の薄い高地でも、内外圧を平衡させつつ、押圧時の素早い圧力変化を発生させることが可能になる。よって、反発力の測定には、たわむ面の曲げ剛性だけを考慮すればよい。後述する押圧補助装置により、目のまわりに本装置を固定させ、操作性を上げる。

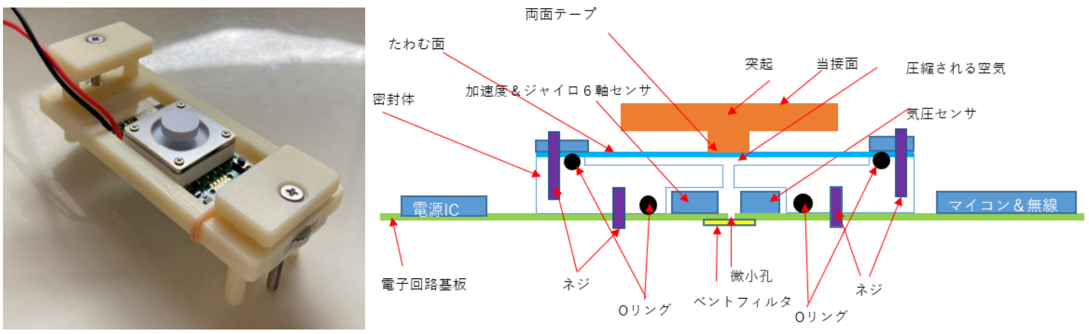

図 2-1. タップアイ眼圧計の外観

2.4.2 使用方法

研究対象者は本機器を自身の眼瞼に押圧することで、眼圧を計測できる。現在でも眼科医は、患者の眼瞼を触診で眼圧を推定することがあり、それに類似する。研究対象者は**図 2-2** のように本機器を手で持ち眼瞼を介して複数回押圧する。眼瞼を Z 軸方向（眼球に対し て垂直）に 2mm から 3mm 押圧する。タップアイを眼瞼に接触させる時間は 1 秒から 2 秒であり、10 回以上押圧する。プログラムが成功と見なした 10 回のデータを採用する。測定全体に要する時間は 30 秒前後である。測定眼は閉瞼した状態で計測するが、反対眼は開瞼するほうが望ましく、反対眼でセンタリングを鏡などで確認しながら行う方が望ましい。また反対側の手で押圧してもよい。眼圧のデータは、本機器のディスプレイに表示される。

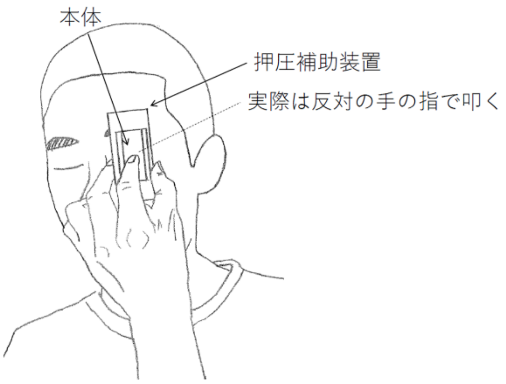

図 2-2. タップアイ眼圧計の測定方法

試験デザインと測定のスケジュールを図 2-3 へ示す。Visit は baseline と 1 か月後の 2 回とする。使用機器は本機器（TET: Tapeye tonometer）の他に既存眼圧計としてゴールドマン Goldmann 圧平眼圧計（GAT）と非接触型眼圧計（NCT）の 2 機種とする。

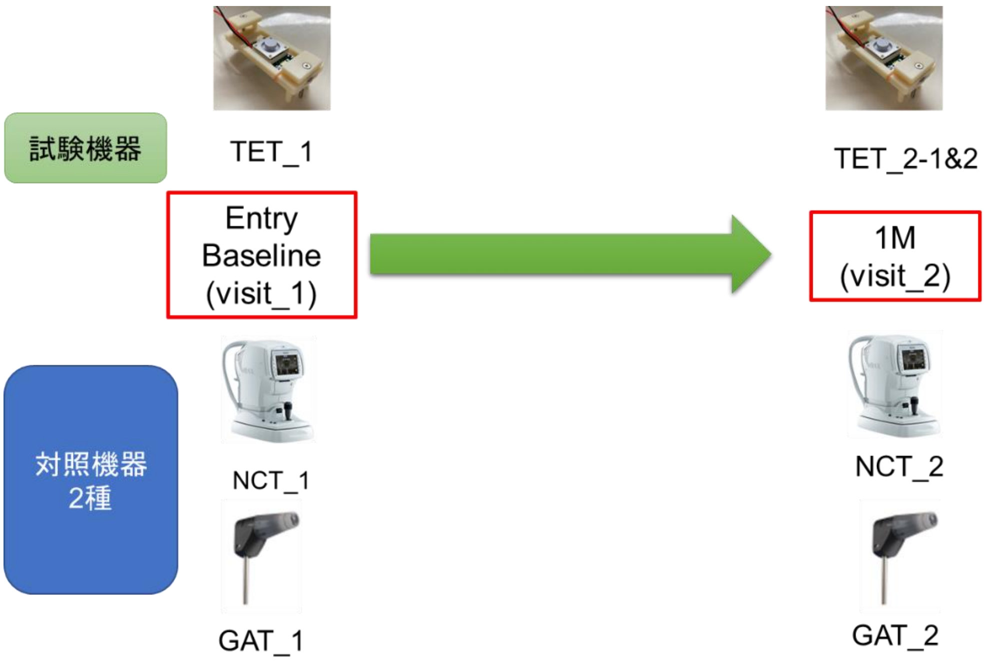

図 2-3. 試験デザインと測定のスケジュール

2.4 試験対象集団

年齢：18 歳以上

性別：制限なし

疾患等：正常眼及び緑内障眼

適格基準：研究責任医師が試験実施可能と判断した症例。

2.5 当該医薬品等の投与等による利益及び不利益

緑内障患者に対しては、本機器を将来的に日常的に使用することで、眼圧のモニタリングが簡便化できて、緑内障治療に対するアドヒアランスが向上することが期待できる。本機器の使用時、眼球押圧による眼の不快感、圧迫感が懸念されるが、押圧回数・押圧距離・時間は最小限に管理されている。但し、本研究の中で安全性評価項目を慎重に検討する。

2.6 準拠する諸規則等

臨床研究法（平成 29 年法律第 16 号）に従い対応する。

2.7 参考データ

これまでに 3D プリンタで制作した様々な硬さのモデル眼（**図 2-4**）を用いて様々な眼圧モデルに対して、本機器が既存眼圧計と正の相関関係を示すことを証明してきた（**図 2-5**)。眼科臨床では、数例の研究対象者を対象として既存眼圧計との比較を試みたところ、眼圧の経時的な増減は一致していた（**図 2-6**)。また各研究対象者における２機種の眼圧値の差異は、5 mmHg 以内であり、本機器の測定精度は認証に耐える結果であった（**図 2-7**)。本機器は眼圧の経時的変化を追跡することが主旨として開発されてきたため、既存眼圧計との相関も重要であるが何より眼圧変動が正確に追えているかが最重要である。そのため本研究では、2 機種における眼圧値の増減・不変の一致率に着目する。

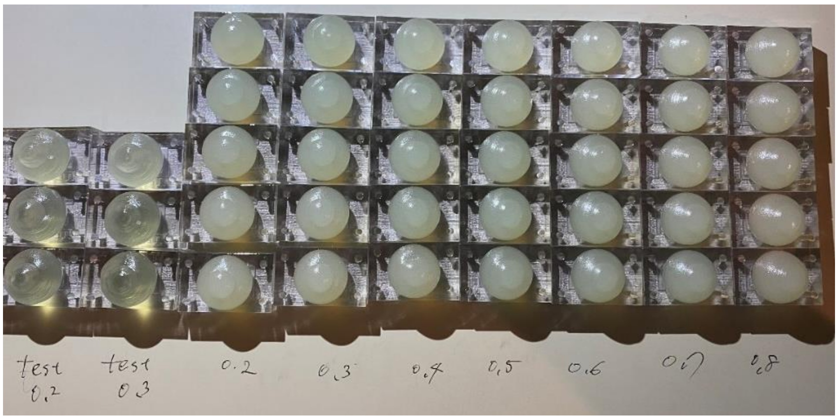

図 2-4. 様々な眼圧を示すモデル眼

実際のヒト眼球に模したデザインを作成し、角膜厚を可変することで異なる眼圧を可能にした。

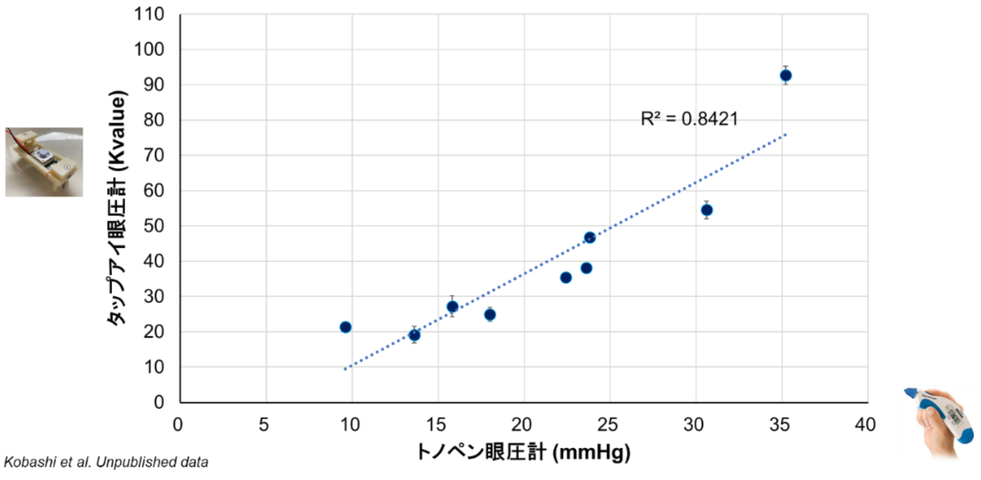

図 2-5. モデル眼を用いた本機器と既存眼圧計の比較：測定精度

良好な相関関係を確認した。

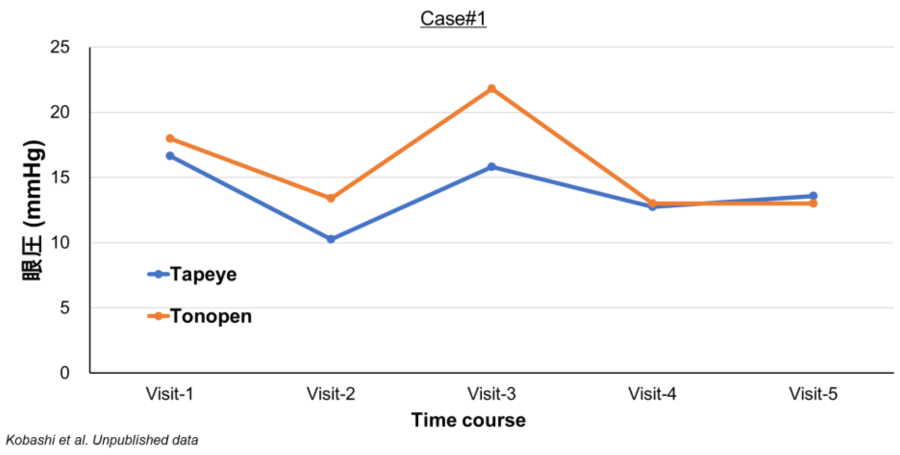

図 2-6.　本機器と既存眼圧計（Tonopen）の眼圧の経時変化を評価した 1 例

2 機種の眼圧の変化はパラレルであった。

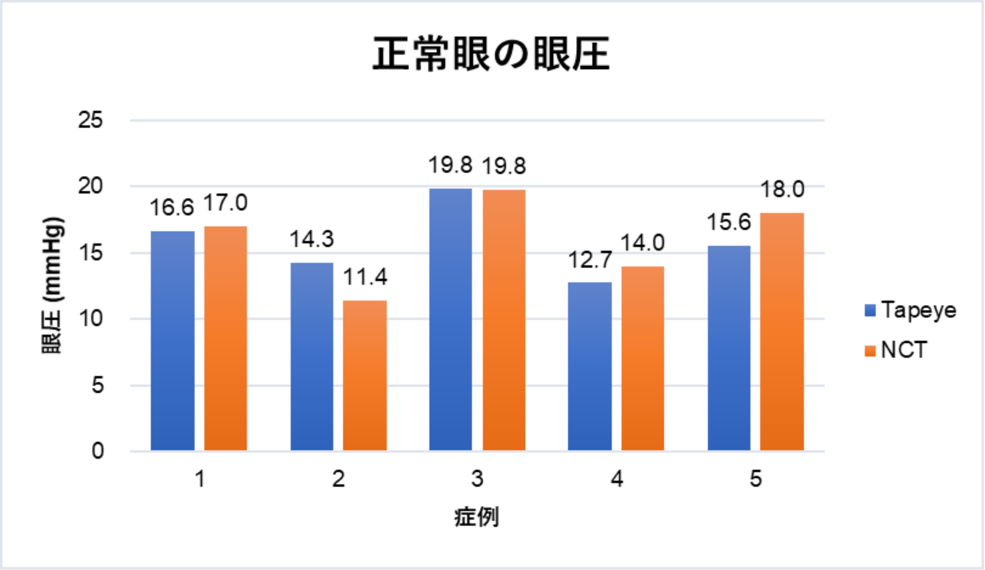

図 2-7.　本機器と既存眼圧計(Non-contact tonometer)の眼圧

５症例において、２機種の眼圧の差異は、5 mmHg 以内であり良好な精度を示した。

3 目的

正常眼及び緑内障眼に対して自己測定眼圧計タップアイを用いた有効性・安全性を検討する臨床研究を計画した。

主要評価項目は本機器と既認証品（GAT と NCT）との間での相関関係を検証することである。主な副次的評価項目としては、本機器を用いた眼圧の経時的変化が正確に追跡できることである。

4 研究デザイン

4.1 評価項目

有効性評価

(1) 主要評価項目

- 本機器と既認証品で求められる眼圧の相関係数の決定係数

(2) 副次的評価項目

- 本機器と既認証品で求められる眼圧の経時的変化の一致率
- 眼圧の繰り返し再現性
- 角膜厚

安全性評価

- 細隙灯顕微鏡
- 皮膚障害（眼周囲の変化）
- 有害事象、研究対象者からの報告

測定機器	評価項目（症例報告書記載）	Entry Baseline (visit_1)	1M (visit_2)
タップアイ眼圧計（TET）	眼圧	○	○x2
ゴールドマンGoldmann 圧平眼圧計（GAT）	眼圧	○	○
非接触型眼圧計（NCT）	眼圧・角膜厚	○	○
細隙灯顕微鏡	眼科学的所見	○	○

図 4-1. 各 Visit の検査項目

4.2 試験方法

本研究は、非無作為化・非盲検試験である。研究対象者は研究機器であるタップアイ眼圧計と対照機器である GAT と NCT を使用する。観察期間は 1 か月として baseline と 1 か月後の 2 回の検査を実施する。組み入れ研究対象者数は 100 例とする。本研究のスケジュールを以下に示す。

<table>
<tr><th></th><th>Visit1
（baseline）</th><th colspan="2">Visit2
（1 か月後）</th></tr>
<tr><td>同意取得</td><td>〇</td><td colspan="2">―</td></tr>
<tr><td>研究対象者情報（年齢、性別）</td><td>〇</td><td colspan="2">―</td></tr>
<tr><td>選択基準・除外基準の確認、症例登録</td><td>〇</td><td colspan="2">―</td></tr>
<tr><td>タップアイ眼圧計（眼圧）※1</td><td>〇
(TET_1)</td><td>〇
(TET_2-1)</td><td>〇
(TET_2-2)</td></tr>
<tr><td>ゴールドマン Goldmann 圧平眼圧系（眼圧）※2</td><td>〇</td><td colspan="2">〇</td></tr>
<tr><td>非接触型眼圧計（眼圧・角膜厚）※2</td><td>〇</td><td colspan="2">〇</td></tr>
<tr><td>細隙灯顕微鏡（眼科学的所見）</td><td>〇</td><td colspan="2">〇</td></tr>
<tr><td>皮膚障害（眼周囲の皮膚）</td><td>〇</td><td colspan="2">〇</td></tr>
<tr><td>有害事象（研究対象者からの報告を含む）</td><td>〇</td><td colspan="2">〇</td></tr>
</table>

※1：プログラムが成功とみなした 10 回のデータを用いるため、眼瞼を 10 回以上押圧する。また、Visit2 の TET_2-1 と TET_2-2 の間は 5 分あける。

※2：1 回のみ測定する。

※3：3 回連続計測した平均値を採用する。

4.3 バイアス

非盲検試験であり懸念事項はない。GAT 計測は研究責任医師が実施するが、その際に他機種の眼圧は開示されないで盲検化されている。

4.4 研究対象者の研究実施及び観察期間

研究実施期間：実施計画の公表日～2023 年 12 月 31 日

4.5 研究機器の管理

本研究で使用するタップアイ眼圧計は施錠可能なスペースで保管する。日常生活において、研究機器の保管環境温湿度の範囲外となることは想定できないが、急激な温湿度変化が生じる場所での保管は避けることとする。

4.6 症例報告書に直接記入され、かつ原資料と解すべき内容の特定

本研究の原資料及び文書を正確かつ適切な時期に作成し、検証可能な記録として適切に保存する。本研究におけるデータの取り扱い及び記録の保存について以下のように定める。

症例報告書は研究責任医師が作成し、記名押印又は署名し、データマネジメント責任者へ提出する。症例報告書の保存期間は、試験終了 5 年を経過した日とする。

症例報告書に記載される原資料となる観察項目は以下の通りである。
- 研究対象者情報（研究対象者 ID, 年齢, 性別）
- 研究対象者としての適格性（選択基準・除外基準の確認）
- 眼圧（3 機種：TET, GAT, NCT）
- 角膜厚
- 細隙灯顕微鏡
- 皮膚障害
- 有害事象、研究対象者からの報告
- 症例登録の中止の有無
- 各 Visit の実施日及び特記事項

4.7 個人情報の取扱い
本研究に従事する者（外部関係者も含む）は、研究対象者の個人情報の保護について適用される「個人情報の保護に関する法律」及び関連通知を遵守する。また、本研究に従事する者は、偽りその他不正の手段により個人情報を取得してはならず、研究対象者の個人情報及びプライバシー保護に最大限の努力を払い、本研究を行う上で知り得た個人情報を正当な理由なく漏らしてはならない（関係者がその職を退いた後も同様とする）。
また、本研究に従事する者は、あらかじめ研究対象者から同意を受けた範囲を超えて、研究の実施に伴って取得された個人情報を取り扱ってはならない。
研究責任医師は、個人情報を取り扱うに当たっては、その利用の目的をできる限り特定し、利用目的の達成に必要な範囲内において、個人情報を正確かつ最新の内容に保たなければならない。また、個人情報の漏えい、滅失又は毀損の防止その他の個人情報の適切な管理のために必要な措置を講じ、当該措置の方法を具体的に実施規定として定める。

4.8 研究対象者のプライバシー保護
本研究において取り扱う研究対象者データは、すべて本研究が独自に付す ID 番号（研究対象者 ID）により管理し、研究対象者の個人情報を保護する。また学会発表及び論文掲載など研究成果の公表時には、検査画像の個人情報マスキングなど、研究対象者のプライバシー保護に万全を期す。本研究で得られた研究対象者に関する個人情報は、研究対象者から同意を得た際の同意説明文書に記載された第三者以外に開示しない。

5 研究対象者の選択・除外・中止基準

5.1 選択基準

研究責任医師が試験実施可能と判断した症例。

本研究にエントリー前に説明同意書に署名にて同意できることとする。

下記の条件を適格基準とする。

・正常眼及び緑内障眼

　＊緑内障研究対象者は、日常の点眼治療を中断する必要はない。

・年齢：18 歳以上

・性別：制限なし

5.2 除外基準

以下のうち 1 つ以上の項目に該当する研究対象者は本研究から除外する：

（ア）眼科手術後 1 か月未満

（イ）臨床研究期間中に眼科の受診が必要となる可能性のある場合

（ウ）その他、研究責任医師が本研究の対象として不適当と判断した場合

5.3 中止基準

本研究の中止基準を下記の様に設ける。

＜症例登録の中止＞

(ア)　細隙灯顕微鏡検査にて研究参加不可能と判断した場合：例：白内障、緑内障の重症化。

用いた水晶体混濁評価：水晶体に新たな混濁が出現した場合

(イ)　皮膚評価：皮膚障害を生じた場合

(ウ)　上記以外の有害事象により本研究の継続が困難と判断した場合

(エ)　研究対象者が中止を申し出た場合、あるいは同意を撤回した場合

(オ)　その他の理由により、研究責任医師が本研究を中止することが適当と判断した場合

研究対象者数を充足させるために、追加登録は行わない。

＜研究実施の中止＞

(ア)　本介入機器であるタップアイ眼圧計によって失明又は失明と同等の視機能低下を認めた又はその疑いがある場合

(イ)　その他の重大な健康被害を認めた又はその疑いがある場合

5.4 やむを得ず、同意の能力を欠く者、同意の任意性が損なわれるおそれのある者を臨床研究の対象者とする場合には、その必然性を明記すること

同意の能力を欠く者、同意の任意性が損なわれるおそれのある者は、本研究の対象外となる。すなわち、研究責任医師は、本研究の対象として不適当と判断する。

6 研究の方法

6.1 研究の方法の内容

本研究は被験機器（TET）と対照機器（GAT と NCT）を介入する非無作為化、非盲検試験である。

6.2 併用療法

本研究に参加する以前から使用している医療機器や薬物については、全て併用は可とするが、3 機種（TET, GAT, NCT）の眼圧計計測時は、自身の眼鏡・コンタクトレンズを外して行う。

6.3 コンプライアンス

本研究における機器使用は、実施施設にて限定的に行うためコンプライアンスは無視できる。

7 有効性の評価

7.1 主要評価項目

<u>他眼圧計と相関係数（決定係数: R^2）</u>

本機器と既認眼圧計（GAT, NCT）と比較した際の相関係数を評価する。その際の評価指標は、既報論文に倣い統計学的に有意な相関関係を示し($p<0.05$)、R^2 ≧0.7 が有効であると設定した（Thrane et al. J Glaucoma. 2020）。

7.2 副次的評価項目

１）<u>眼圧の経時的変化の一致率</u>

本研究期間に研究対象者は、異なる日時に 2 回計測することになるが、本機器と既認眼圧計（GAT）で求められる眼圧変化（ΔIOP）が両機種で一致することが有効性の指標とする。一致の定義は± 2 mmHg の変化を基準とする。

１例を示す。既認証品で計測した際に baseline から 1 か月で 10 mmHg から 15 mmHg に変化したとする（5 mmHg 増加）。本機器では 12 mmHg から 19 mmHg に変化した（7 mmHg 増加）。± 2 mmHg の変化で増減・不変という定義をした場合、先述の 1 例は 2 機種とも増加したことを示す。眼圧値の増減・不変の一致率を評価項目として、目標は全体の 80% 以上を目指す。

２） <u>同一研究対象者における繰り返し再現性</u>
本機器の繰り返し再現性は測定の信頼性において重要である。Visit 2（1 か月後）において研究対象者は本機器を 5 分間あけて 2 回測定を行う（TET_2-1&TET_2-2）。TET_2-1 と TET_2-2 の測定誤差は、級内相関係数（ICC）を求めて評価するが、0.8 以上を目標とする。

*本研究では角膜厚は NCT を用いて求める。一般的に角膜厚と眼圧は正の相関関係を示すため、角膜厚の評価は眼圧に影響する。

7.3 有効性評価指標の評価、記録及び解析方法
有効性評価指標は全て症例報告書に明記する。研究責任医師は本研究期間中の Visit（baseline と 1 か月後）が終えた後、データマネジメント責任者に連絡し、データ入力・管理を依頼する。

8 安全性の評価

8.1 安全性評価指標
安全性評価項目は本研究機器を使用した研究対象者に発現した全ての好ましくない医学的事象であり、本研究機器、医薬品との因果関係の有無は問わないものとする。
- 細隙灯顕微鏡
- 皮膚障害（眼周囲の皮膚）
- 有害事象、研究対象者からの報告

8.2 安全性管理体制
研究実施中、研究責任医師は、研究対象者の来院日では診療及び検査により、来院日以外では研究対象者との緊急連絡方法を確保し、常に研究対象者の健康状態を把握する。
有害事象が発現した場合には、必要に応じて研究対象者への適切な医療の提供を行い、研究対象者の研究中止など、研究対象者の安全を確保する。

8.3 安全性評価指標の評価、記録及び解析方法

研究責任医師は、観察期間終了まで安全性評価指標を症例報告書に明記して保存する。データの統計学的解析は、統計解析責任者が行う。定性化できるパラメータ（細隙灯顕微鏡、皮膚障害、有害事象）はそのまま評価する。

8.4 疾病等

疾病等とは、本研究の実施に起因するものと疑われる疾病、障害若しくは死亡又は感染症に加え、臨床検査値の異常や諸症状をいう。有害事象とは、本機器を用いて眼圧計測した際に研究対象者に生じたあらゆる好ましくない医療上の出来事をいう。必ずしも、眼圧計測と因果関係が明らかなもののみを示すものではない。つまり有害事象とは、眼圧計測を実施した際に生じる、あらゆる好ましくないあるいは意図しない徴候、症状又は病気のことであり、本機器との因果関係の有無は問わない。なお、症状及び徴候が本機器使用後に悪化した場合は、新たな有害事象として取り扱う。

重篤な有害事象とは、有害事象のうち、下記の①～⑤に該当するものをいう。

① 死に至るもの

② 生命を脅かすもの

「生命を脅かす」とは、その事象が起こった際に、研究対象者が死の危険にさらされていたという意味である。

③ 治療のために入院又は入院期間の延長が必要となるもの

「治療のために入院」とは、有害事象のために、研究対象者が医療機関に 1 日以上入院する場合をいう。一方で、本研究開始前の状態から悪化していない原疾患又は合併症に対する検査や処置等のための入院、有害事象の治療を目的としない社会的及び便宜的入院、本研究参加開始前より予定していた治療又は検査を実施するための入院は、「治療のために入院」に該当しない。

④ 永続的又は顕著な障害・機能不全に陥るもの

⑤ 医学的に重篤と考えられる事象

研究責任医師は、認定臨床研究審査委員会及び厚生労働大臣へ報告する。報告に際して、研究対象者等への説明及び治療等、必要な措置を講じる。

1. 予測できない死亡、死亡につながるおそれのある疾病等

報告先：認定臨床研究審査委員会及び厚生労働大臣　　　　　報告期限：7 日以内

2. 死亡、死亡につながるおそれのある疾病等

報告先：認定臨床研究審査委員会　　　　　　　　　　　　　報告期限：15 日以内

3. 次に掲げる疾病等のうち、予測することができないもの

(1) 治療のために医療機関への入院又は入院期間の延長が必要とされる疾病等

(2) 障害

(3) 障害につながるおそれのある疾病等

(4) 死亡又は上記に準じて重篤である疾病等

(5) 後世代における先天性の疾病又は異常

報告先：認定臨床研究審査委員会及び厚生労働大臣　　報告期限：15 日以内

4. 上記以外のもの

報告先：認定臨床研究審査委員会　　報告期限：定期報告時

有害事象の評価は以下の手順で行う。

事象名：有害事象の事象名を特定する。誘因となる疾患名が特定される場合は、個々の症状ではなくその診断名を事象名とする。

発現日：有害事象が発現した日又は発現を確認した日を発現日とする。

重症度：有害事象の重症度を以下の３段階で評価する。

１．軽度：不快感を覚えるが、日常生活に支障はない

２．中等度：日常生活に制限又は影響がある程度に不快である

３．高度：仕事や日常生活を行うことができない

発現した有害事象に対して実施した処置（薬物治療又はそれ以外の処置）の有無、有の場合はその内容を記録する。

8.5 疾病等発生後の臨床研究の対象者の観察期間

以下に示す手順に従い、疾病等及び有害事象の追跡調査を行う。

なお「回復」とは、評価期間に発現した有害事象がもとの状態に復すること、本研究開始時に存在した疾患、症状及び徴候が本研究終了後に悪化した場合の有害事象では、本研究開始前に状態に復することをいう。

１．評価期間終了日又は中止日に有害事象が回復していない場合、研究責任医師は該当する研究対象者に追跡調査の趣旨を説明し、4 週間後（以内）に追跡調査を行う（その実施日を追跡調査日とする）。

２．追跡調査日に有害事象が回復していない場合、本研究との関連性が否定できない有害事象については、可能な場合その追跡調査日以降もその有害事象が回復又は安定するまで調査を継続する。本研究と関連性が否定出来る場合は、前項の追跡調査により、調査を終了する。

３．観察期間終了日又は中止日から追跡調査日までの間に、本研究との関連性が否定できない新たな重篤な有害事象が発現した場合及び観察期間終了日又は中止日に回復していない本研究と関連性が否定できない有害事象が「重篤な有害事象」に該当するようになった場合は、追跡調査日以降もその重篤な有害事象が回復又は安定するまで追跡調査を行う。

8.6　不具合の報告

臨床研究法施行規則第 55 条に基づき、実施する研究責任医師は当該特定臨床研究に用いるタップアイ眼圧計の不具合によって有害事象が発生した場合、患者の健康状況や、タップアイ眼圧計の不具合状況を実施医療機関の管理者、当該製品の製造販売会社に速やかに報告しなければならない。また、発生した不具合が「疾病等」に該当する場合は、「8.4 疾病等」の手順に従って報告する。

9 統計解析

9.1 解析方法

他眼圧計と相関係数（決定係数: R^2）

本機器と既認眼圧計（GAT, NCT）と比較した際の相関係数（R^2）を評価する。

眼圧の経時的変化の一致率

本研究期間に研究対象者は、異なる日時に 2 回計測することになるが、本機器と既認眼圧計（GAT）で求められる眼圧変化（ΔIOP）が両機種で一致することが有効性の指標とする。一致の定義は± 2 mmHg の変化を基準とする。

同一研究対象者における繰り返し再現性

TET_2-1 と TET_2-2 の測定誤差は、級内相関係数（ICC）を求めて評価する。

9.2 登録症例数

・最小必要サンプルサイズは 4 例であった。

下記条件で計算した。

- αエラー（Type I）　：0.05
- βエラー（Type I）　：0.80
- R^2　：0.70

9.3 有意水準
各評価項目における検定での有意水準は両側 5%とする。

9.4 試験中止基準
8.4 に示す重篤な有害事象が生じた場合は、登録症例数が予定症例数に達しない時点であっても、試験中止とする。

9.5 試験データの取り扱い
中止、脱落又は逸脱等の理由により、Visit 2 までのデータに欠測値が生じた場合、データの補完は実施しないものとする。
中止及び脱落時のデータについては、集計及び解析上の取り扱いを以下のように規定する。
- 規定来院時に中止又は脱落した場合：当該規定来院時のデータとして集計及び解析
- 規定来院時以外で中止又は脱落した場合：次回来院予定時のデータとして集計及び解析

9.6 当初の統計的な解析計画を変更する場合の手順
・ 当初の統計的な解析計画からの変更がある場合は、研究計画書を改訂し、臨床研究の総括報告書においても説明する。

9.7 解析対象集団
本研究の主目的は本機器の有効性・安全性を確認することである。本研究における解析対象集団を以下のように定義する。
① 安全性評価対象集団
研究対象者の同意を取得した症例のうち以下の症例を除く集団
・baseline 検査前に同意を撤回した症例
・本機器タップアイ眼圧計を一度も使用していない症例

② 有効性評価対象集団（FAS）
研究対象者の同意を取得した症例のうち以下の症例を除く集団
・baseline 検査前に同意を撤回した症例
・本機器タップアイ眼圧計を一度も使用していない症例
・有効性評価に係るデータを有していない症例

③ 有効性評価対象集団（PPS）
FAS 群のうち、以下の症例を除く集団
・baseline 検査後に本研究への参加を中止したもしくは同意を撤回した症例
・本機器タップアイ眼圧計の使用方法及び使用頻度を本計画書から逸脱した症例

10 モニタリング

研究対象者に関する本研究関連データは、症例報告書に記録する。研究責任医師は、データが正確に記録されていることを確認し、症例報告書への記名押印もしくは署名する。モニターは研究対象者の安全性と権利が保護されていること、承認された最新の研究計画書及びその他の本研究に関する同意書、並びに適用されるすべての規制要件を遵守して本研究が実施されていることを確認する。

11 倫理的な配慮

11.1 研究対象者の利益と不利益と副作用
利益：
本研究の該当研究対象者は、タップアイ眼圧計を用いることで低侵襲的に簡便に眼圧を計測できる利益を共有できる。

不利益：
予想される不利益を下記に述べる。
・タップアイ眼圧計の長期的有効性・安全性は不明であること。

副作用：
本研究による有害事象（副作用）の発現リスクは以下の通りである。
・眼瞼の圧迫感
・眼球の刺激感

11.2 インフォームド・コンセント
本研究に係る業務を行う前に認定臨床研究審査委員会により承認を受けた同意説明文書を用いて研究対象者から書面により同意を取得する。研究対象者の同意取得後から本研究への参加期間中に、本研究への参加に影響を及ぼす情報が新たに得られた場合には、同意説明文書の改訂及び速やかに該当する情報を研究対象者に提供し、本研究への参加継続に関

して可否を確認する。認定臨床研究審査委員会より改訂版の同意説明文書の承認を取得した後、当該同意説明文書を用いて再度、研究対象者から書面により同意を取得する。

研究対象者が著しい視覚障害を伴う場合は、研究責任医師は、同意説明文書を渡す他、口頭で全て説明し、研究対象者が理解できるようにする。その上で、研究対象者が十分に理解できない場合、研究責任医師は研究対象者の組み入れ行わない。

11.2.1 代諾
選択基準が 18 歳以上であるため代諾者による同意は必要としない。

11.2.2 インフォームド・アセント
選択基準が 18 歳以上であるため必要としない。

11.3 研究に関する情報公開
本研究は、厚生労働省が整備するデータベース（以下「jRCT」（Japan Registry of Clinical Trials）という。）に記録し、公表する。

11.4 研究対象者及びその関係者からの相談等への対応
研究責任医師が対応する。説明文書に当該医師の所属先の直通番号を明記した。

11.5 利益相反
本研究はタップアイ眼圧計を開発・製造している株式会社トニジから実施に必要な資金、資材等の提供を受けて実施する。資金等の詳細については、実施医療機関と株式会社トニジとの研究の委受託契約書に定める。
研究責任医師は、実施医療機関の利益相反の管理に関する規定に準じ、 利益相反管理計画書を作成し、認定臨床研究審査委員会の審査を受ける。本研究結果の学会発表や論文公表に際しては、研究資金に関して公表し、透明化を図る。

11.6 試験終了後の研究対象者への対応
通常、各研究対象者は visit 2 後に試験終了となる。緑内障研究対象者は、日常診療を継続する。本研究参加に際して、点眼治療を中断する必要がない。

11.7 遺伝的特徴等に関する重要な知見への対応
該当なし

12 データの取り扱いと記録保存

12.1 データの取り扱いと記録保存

本研究の症例報告書を正確かつ適切な時期に作成し、適切に保存する。本研究におけるデータの取り扱い及び記録の保存について以下のように定める。

・研究責任医師が作成した症例報告書の様式を用いて、実施医療機関にて記載できるものとする。研究責任医師は、症例報告書の記載内容に問題がないことを確認した症例報告書に記名押印又は署名し、データマネジメント責任者へ提供する。又、症例報告書の写しを実施医療機関に保管する。

・データマネジメント責任者は、入力・修正・確認が終了した Excel 形式のデータ入力用ファイルを最終生成物として CD-R に記録し、研究責任医師に報告する。具体的には、データマネジメント担当者２名がそれぞれ別の Excel ファイル(Microsoft 社 Excel で作成されたデータ入力用ファイル)に、紙媒体 CRF（症例報告書）に記載のデータ入力を行い、入力済みの２つの Excel ファイルを比較する。比較が一致しなかった場合、正確なデータを入力し、誤入力を修正する（ダブルデータエントリー）。業務担当者間において、ファイルの比較、確認、入力ミスの修正を行う。また、データの改ざん等の防止のため、データの修正等があれば修正履歴を別ファイルで記録、管理を行う。電子化した後は、症例報告書は破棄する。

・実施医療機関の研究責任医師におけるすべての記録の保存期間は、本研究終了後 5 年を経過した日までとする。廃棄対象となる症例報告書はシュレッダーの上、廃棄する。電子データは完全に消去する。

12.2 データの将来の別研究での利用及び他機関への提供の可能性

・本研究において取り扱う症例報告書以外の研究対象者データは、すべて本研究が独自に付す ID 番号（研究対象者 ID）を用いて管理する。別研究へのデータ利用及び他機関データ共有は可能とするが、研究対象者の個人情報はマスキングされたデータとし、研究責任医師が全ての責任を負う。

13 研究対象者の経済的負担及び保険その他の措置

・研究対象者は、本研究の参加に際して、一切の経済的負担を負わない。また、本研究への参加による研究対象者の負担を軽減するため、負担軽減費（QUO カード 10,000 円券）を提供する。
・万一、試験期間中に健康被害が発生した場合、研究対象者に対して無償で適切な治療を行う。また、死亡や後遺障害といった重篤な健康被害が発生した場合には、研究責任医師が加入する臨床研究保険より補償金が支払われる。・本研究終了後のフォローアップは、従来通り保険診療に切り替えて行う。

14 臨床研究に関する情報の公表に関する取決め

・本研究で提供又は収集される情報はいずれも研究責任医師に帰属する。
・研究責任医師は、本研究の総括報告書の概要並びに総括報告書を jRCT に公開すると共に、結果を学会発表や論文として報告すること等により、情報公開する。

15 補遺

特記事項無し。
