# Supplementary material for: Evaluation of a new transpalpebral tonometer for self-measuring intraocular pressure
Source: PLoS One. 2024 May 15;19(5):e0302568. doi: 10.1371/journal.pone.0302568 (PMC11095731; doi:10.1371/journal.pone.0302568)
Supplement: S2 File — (PDF) [file pone.0302568.s003.pdf]

Exploratory study of intraocular pressure changes in the new self-measuring tonometer "tap eye" in normal and glaucoma patients

Research Protocol

Implementing medical institution: Medical Corporation Jinjikai Yokohama Tsurumi Central Eye Clinic
Principal investigator: Teshigawara Go
Medical institution address: 1-2-1 Tsurumi Chuo, Tsurumi Ward, Yokohama City, Kanagawa Prefecture 230-0051

Scheduled research period: From the date of publication of the implementation plan to December 31 , 2023

Version number: Version 1.0
Creation Date : July 1 , 2022

List of abbreviations and definitions

Abbreviations	full expression
IOPs	Intraocular pressure
NCT	Non-contact tonometer
GAT	Goldmann applanation tonometer
TET	Tapeye tonometer _

the term	definition

table of contents

Summary 4
1 Implementation system 5
2 Background Information 6
3 purpose 143
4 study design 143
5 Criteria for inclusion , **exclusion, and discontinuation of research subjects16** 18**of efficacy17** 20

wrap up

0.1 schema

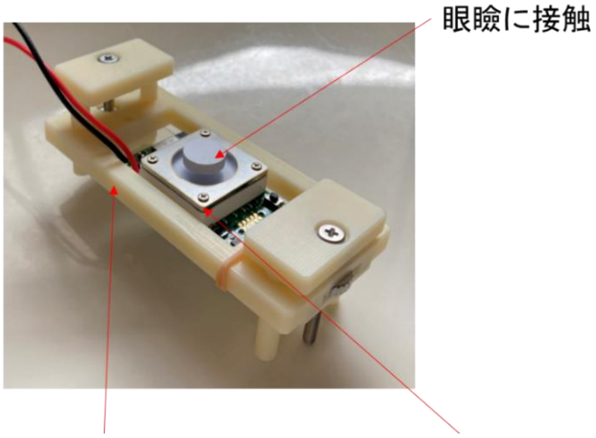

0.2 Purpose

To investigate the measurement accuracy and reproducibility of the self-monitoring tonometer Tap Eye for normal eyes and glaucomatous eyes.

0.3 target

It is intended for normal eyes and glaucomatous eyes.

0.4 test treatment

No therapeutic intervention.

0.5 Planned number of registrations and research period

Total 100 cases, from date of publication of implementation plan to December 31 , 2023

0.6 Contact

1-2-1 Tsurumi Chuo, Tsurumi Ward , Yokohama City, Kanagawa Prefecture 230-0051
Medical Corporation Jinjikai Yokohama Tsurumi Central Eye Clinic
Akira Kitamura
Email : ts urumi.chuoh.eye.cs@gmail.com _

1 Implementation system

1.1 Research information
Exploratory study of intraocular pressure change of new self-monitoring tonometer Tap Eye in normal subjects and glaucoma patients

Accredited Clinical Research Review Committee: Medical Corporation Shinanokai Shinanozaka Clinic Clinical Research Review Board

1.2 Principal Investigator
Name: Teshigawara Go
Affiliation: Medical Corporation Jinjikai Yokohama Tsurumi Central Eye Clinic
Affiliation: Medical Office
Position: Doctor
address: 1-2-1 Tsurumi Chuo, Tsurumi Ward , Yokohama City, Kanagawa Prefecture 230-0051
Phone number: 045-508-1017
Email : teshitake@gmail.com

1.3 Responsible person for data management
Name: Rina Kawasaki
Affiliation: Nagasaki University Hospital
Affiliation: Clinical Research Center
Position: Data Manager
Address: 1-7-1 Sakamoto , Nagasaki City, Nagasaki Prefecture 852-8501
Phone number: 095-819-7726
Email : kawasaki@nagasaki-u.ac.jp

1.4 Person responsible for statistical analysis
Name: Mizukami Takahiro
Affiliation: Tane Memorial Eye Hospital (scheduled to start in April 2023)
Affiliation: Ophthalmology
Position: Doctor
Address: 1-1-39 Sakaigawa, Nishi-ku , Osaka-shi, Osaka 550-0024
Phone number: 06-6581-5800

1.5 Person responsible for monitoring
Name: Junko Fujii
Affiliation: New Drug Research Center Co., Ltd.
Affiliation: Research Division Clinical Research Department
Position: General Manager
Address: 1-7-1 Yurakucho, Chiyoda- ku , Tokyo 100-0006 Yurakucho Denki Building South Building 4F
Phone number: 03-6551-2335
Email : j-fujii@ndrcenter.co.jp

1.15 Responsible organization for supporting research and development plans
Name: Yumi Natani
Affiliation: UMIN Co., Ltd.
Affiliation: Representative Director
Address : 1-2-6 Tsuruma , Machida City, Tokyo 194-0004
Phone number: 042-795-4812
Email : y-nata@umin.co.jp

2 Background information

2.1 Status of target diseases

Glaucoma is defined as a disease characterized by functional and structural abnormalities of the eye with characteristic changes to the optic nerve and visual field, and which may be ameliorated or prevented by sufficient reduction of intraocular pressure. (Japan Glaucoma Guidelines). Glaucoma is one of the leading causes of blindness in Japan. In a detailed glaucoma epidemiological survey conducted from 2000 to 2002 (Iwase et al. Tajimi Study. Ophthalmology. 2004) , the prevalence of glaucoma in Japanese aged 40 years and over was 5.0 %, and the 2016 estimate was The number of patients is 4.65 million. In glaucoma, since the disability gradually progresses without the patient's awareness, it is important to prevent or suppress the progression of the disability through early detection and early treatment. Lowering the intraocular pressure is considered the only treatment for glaucoma, and eye drops are used, but intraocular pressure fluctuates throughout the day, making it difficult to apply eye drops at appropriate times. We want to know the intraocular pressure all the time, but patients do not have frequent access to medical institutions, and it is not always possible to know the intraocular pressure. If patients can easily and safely measure intraocular pressure at home, (1) it will be easier to evaluate the treatment effect and their medication adherence will improve. (2) Contribute to further elucidation of the pathology of glaucoma. (3) Medical costs can be reduced if eye drops that lower intraocular pressure are used only when necessary (when intraocular pressure increases).

2.2 Existing tonometer

Below is a list of tonometers currently certified in Japan.

眼瞼を通して自己測定

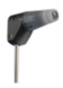	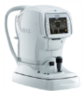	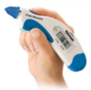	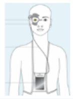	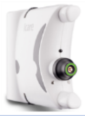	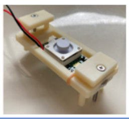

	Goldmann	ノンコン	トノペン	Triggerfish	iCareHome	タップアイ
接触	角膜	非接触	角膜	角膜	角膜	眼瞼
携帯性	×	×	○	○	○	◎
麻酔	要	不要	要	不要	不要	不要
侵襲	有り	少ない	有り	有り	少ない	無
精度	高い	やや低い	低い	やや低い	やや高い	やや高い
売価(円)	15万	100万	50万	5万円/CL1枚	100万円	未定
企業名	HAAG-STREIT (独)	Nidek (日)	Reichert (米)	SENSIMED (スイス)	icare (フィンランド)	弊社
特徴	ゴールドスタンダード	スクリーニング	携帯可能	付属品多い	自宅で可能	瞼を介して低侵襲 低価格
分類	クラスⅡ	クラスⅡ	クラスⅡ	クラスⅢ	クラスⅡ	新名称?
自己計測	×	×	×	△	○	◎
遠隔医療	×	×	×	×	×	○

2.3 Current issues, unclear points, etc. that lead to the need for clinical research

iCare Home is the only tonometer on the market that allows end users to self-monitor . As shown in the table , the disadvantages of iCare Home are: 1) it requires some tricks for self-monitoring, 2) it is expensive at 1 million yen per device , and 3) the probe directly hits the cornea, which makes it difficult for the patient. Fear-mongering, etc. This device tap eye can solve these problems. Since early detection and early intervention are important to avoid blindness in glaucoma, the use of this device facilitates monitoring of intraocular pressure data. In addition, since the price is set so that each patient can own one unit, the adherence of the eye drops is improved, and the effect after the eye drops can be felt. Ultimately, it can contribute to suppressing the increase in medical expenses for glaucoma treatment.

2.4 The following information on the relevant research equipment used in clinical research

2.4.1 Research equipment

self-monitoring tonometer tap eye

MEMS (Micro Electro Mechanical Systems) are components in which electronic circuits, sensors, and actuators that move mechanically are built on silicon wafers. Typical applications for MEMS are automobiles and smartphones. This device is a tonometer that measures the amount of change and repulsive force due to pressure through the eyelid using multiple MEMS sensors and a special case and displays it as an intraocular pressure value. At present, tonometers using MEMS

sensors are not commercially available. What is common to the operating principles of existing tonometers is that the repulsive force with respect to the amount of deformation of the object to be measured is measured . As shown in **Figure 2-1 ,** this device consists of an acceleration sensor to know the amount of deformation (the double integral of acceleration is distance) , an air pressure sensor to know the repulsive force, and a sealed space that bends only on one side (internal pressure and repulsive force are one-to-one correspondence) , a gyro sensor that corrects the direction of gravity due to rotation (the same IC package as the acceleration sensor), and a vent filter that allows for slow ventilation even if the outside air pressure changes so that the difference in inside and outside air pressure can be ignored. Become. The intraocular pressure is determined by applying a correction formula to the ratio of the amount of deformation and the repulsive force. A cushioning material is placed on the lower surface, and the main body is pressed in the direction of the eyelid by lightly tapping. A gyro sensor corrects the change in the direction of gravity due to unnecessary rotation. A vent filter (membrane that allows air to pass through but water does not pass) with an appropriate amount of ventilation makes it possible to generate a rapid pressure change during pressing while balancing the internal and external pressure even at high altitudes where the air is thin. Therefore, only the flexural stiffness of the deflecting surface needs to be considered in measuring the repulsive force. A press assisting device, which will be described later, is used to fix the device around the eye and improve operability.

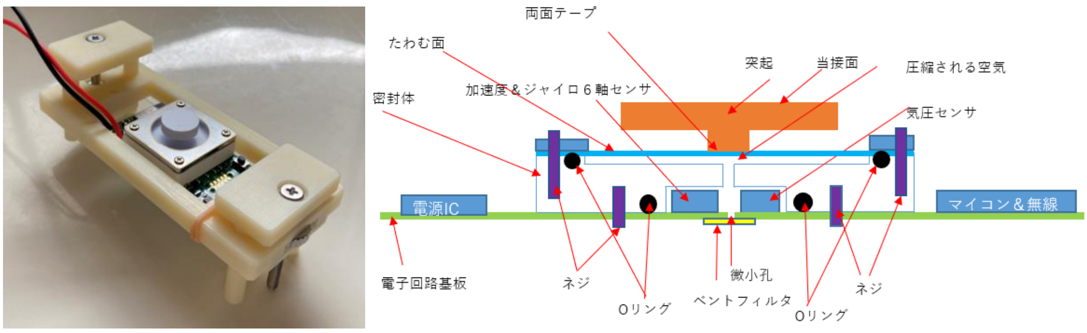

Figure 2- 1. Appearance of tap eye tonometer

2.4.2 Usage

Research subjects can measure intraocular pressure by pressing the device against their own eyelids. Ophthalmologists still sometimes estimate intraocular pressure by palpating a patient's eyelids, and are analogous. The research subject holds the device by hand and presses it multiple times through the eyelid as shown in **Figure 2-2 .** Press the eyelid 2 to 3 mm in the Z- axis direction (perpendicular to the eyeball) . The time for which the tap eye is brought into contact with the eyelid is 1 to 2 seconds, and it is pressed 10 times or more. Adopt the data of 10 times that the program considers successful. The time required for the entire measurement is around 30 seconds. The eye to be measured is measured with the eyelid closed, but it is preferable to open the eyelid of the opposite

eye, and it is preferable to perform the measurement while checking the centering of the opposite eye with a mirror or the like. Moreover, you may press by the hand of an opposite side. Intraocular pressure data are displayed on the device display.

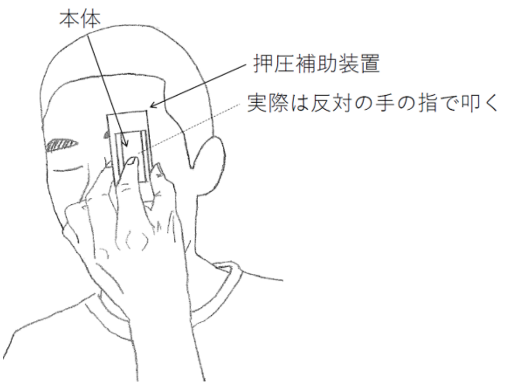

Figure 2-2.Tap eye tonometer measurement method

The study design and measurement schedule are shown in Figure 2-3 . Two visits , one at baseline and one month later . In addition to this device (TET: Tapeye tonometer), the equipment used is Goldmann Goldmann as an existing tonometer There are two models: an applanation tonometer (GAT) and a non-contact tonometer (NCT) .

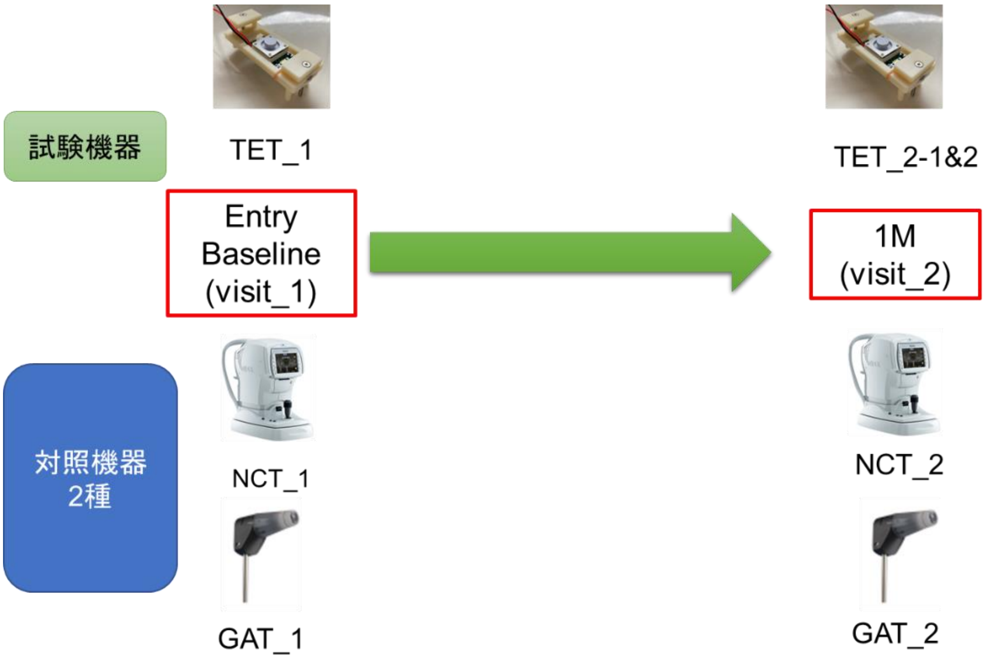

Figure 2-3 . Study design and measurement schedule

2.4 Study Population

Age : 18+

Gender: no restrictions

Diseases, etc.: normal eyes and glaucomatous eyes

Eligibility criteria: Cases judged by the principal investigator to be feasible for the study.

2.5 Advantages and disadvantages due to administration, etc. of the drug, etc.

For glaucoma patients, future routine use of this device is expected to simplify intraocular pressure monitoring and improve adherence to glaucoma treatment. When using this device, there is concern about eye discomfort and oppressiveness due to eyeball pressure, but the number of times of pressure, pressure distance, and time are managed to a minimum. However, safety endpoints will be carefully considered in this study.

2.6 Applicable regulations, etc.

Respond in accordance with the Clinical Research Act (Law No. 16 of 2017).

2.7 Reference data

So far, we have demonstrated that this device shows a positive correlation with existing tonometers for various intraocular pressure models using model eyes with various hardnesses (**Fig . 2-4)** produced with a 3D printer. (**Figure 2-5**).

In clinical ophthalmology, when a comparison with existing tonometers was attempted on several research subjects, changes in intraocular pressure over time were consistent (Fig. **2-6)** . In addition, the difference in intraocular pressure values between the two models for each research subject was within 5 mmHg , and the measurement accuracy of this device was certified (**Fig. 2-7**). Since this device was developed with the aim of tracking changes in intraocular pressure over time, it is important to have a correlation with existing tonometers, but above all, it is most important to accurately track changes in intraocular pressure. Therefore, in this study, we focus on the rate of agreement between changes in intraocular pressure values and invariance between the two models.

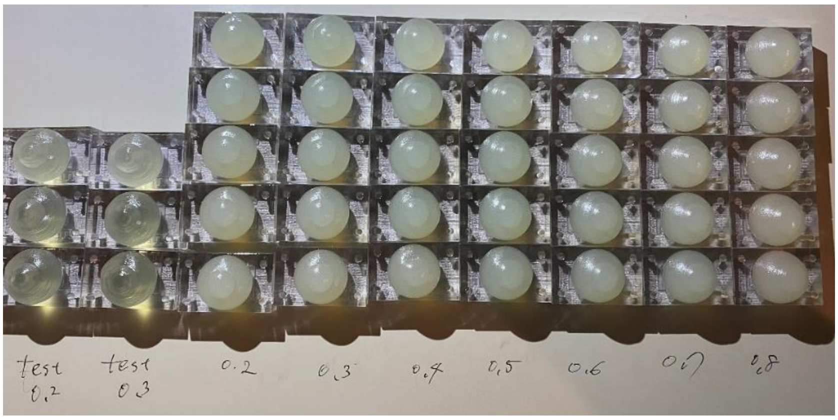

Figure 2-4 . Model eye showing various intraocular pressures

We created a design that mimics the actual human eyeball and enabled different intraocular pressures by varying the corneal thickness.

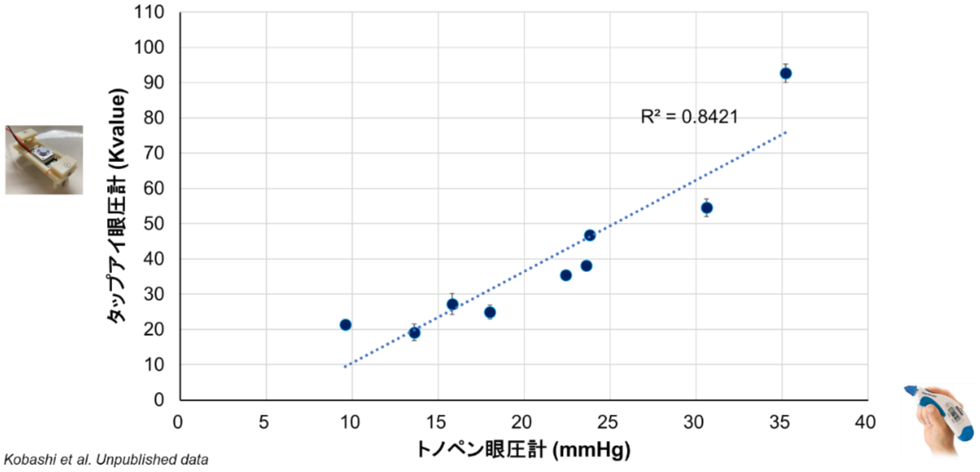

Figure 2-5 . Comparison between this device and an existing tonometer using a model eye: measurement accuracy

A good correlation was confirmed.

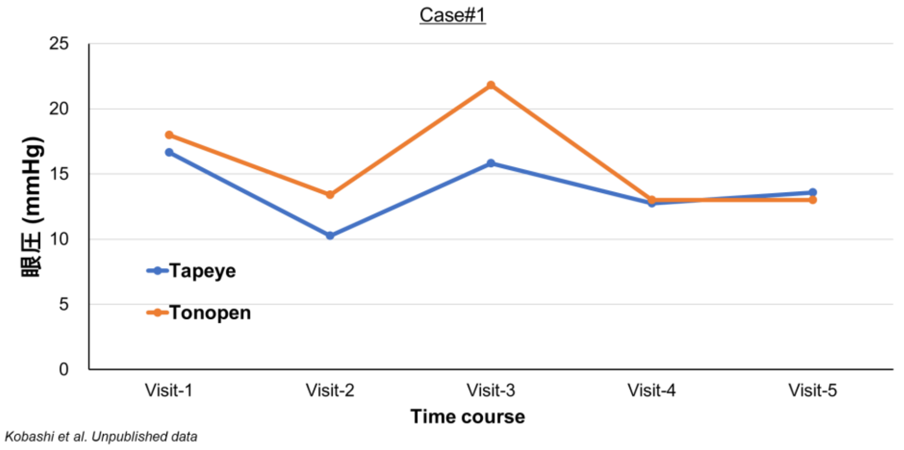

Figure 2-6 . A case of evaluating changes in intraocular pressure over time between this device and an existing tonometer (Tonopen)

two models were parallel.

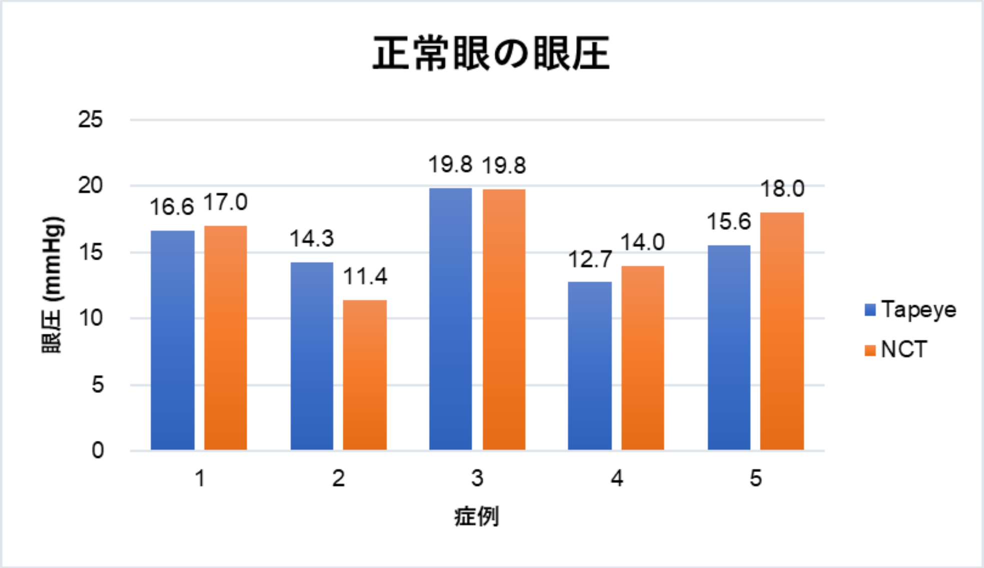

Figure 2-7 . Intraocular pressure between this device and existing non-contact tonometer

In five cases, the difference in intraocular pressure between the two models was within 5 mmHg , demonstrating good accuracy.

3 purposes

A clinical study was planned to examine the efficacy and safety of self-monitoring tonometer Tap Eye in normal and glaucomatous eyes.

The primary endpoint is to verify the correlation between this device and the certified products (GAT and NCT). The main secondary endpoint was the ability to accurately track changes in intraocular pressure over time using the device.

4 Study design

4.1 Evaluation items

Efficacy evaluation

(1) Primary endpoint

-Coefficient of determination of intraocular pressure correlation coefficient required between this device and certified products

(2) Secondary endpoints

- Concordance rate for changes in intraocular pressure over time required for this device and certified products
- Repeatability of intraocular pressure
-corneal thickness

Safety evaluation

-Slit lamp microscope
- Skin disorders (periocular changes)
- Adverse events, reports from study subjects

測定機器	評価項目（症例報告書記載）	Entry Baseline (visit_1)	1M (visit_2)
タップアイ眼圧計（TET）	眼圧	○	○x2
ゴールドマンGoldmann 圧平眼圧計（GAT）	眼圧	○	○
非接触型眼圧計（NCT）	眼圧・角膜厚	○	○
細隙灯顕微鏡	眼科学的所見	○	○

Figure 4-1. Inspection items for each Visit

4.2 Test method

This study is a non-randomized, open-label study. Study subjects will use the study instrument, the tap-eye tonometer, and the control instruments, GAT and NCT . The observation period is 1 month, and the baseline and 1 month later tests are performed twice . The number of enrolled study subjects is 100 . The schedule for this research is shown below.

<table>
<tr><th></th><th>Visit 1
(baseline)</th><th colspan="2">Visit2
(1 month later)</th></tr>
<tr><td>Consent acquisition</td><td>○</td><td colspan="2">-</td></tr>
<tr><td>Research subject information (age, gender)</td><td>○</td><td colspan="2">-</td></tr>
<tr><td>Confirmation of inclusion/exclusion criteria, case registration</td><td>○</td><td colspan="2">-</td></tr>
<tr><td>Tap eye tonometer (intraocular pressure) *1</td><td>○
(TET_1)</td><td>○
(TET_2-1)</td><td>○
(TET_2-2)</td></tr>
<tr><td>Goldmann G oldmann applanation intraocular pressure system (intraocular pressure) *2</td><td>○</td><td colspan="2">○</td></tr>
<tr><td>Non-contact tonometer (intraocular pressure/corneal thickness) *2</td><td>○</td><td colspan="2">○</td></tr>
<tr><td>Slit lamp microscope (ophthalmological findings)</td><td>○</td><td colspan="2">○</td></tr>
<tr><td>Skin disorders (periocular skin)</td><td>○</td><td colspan="2">○</td></tr>
<tr><td>Adverse events (including reports from study subjects)</td><td>○</td><td colspan="2">○</td></tr>
</table>

* 1 : The program uses the data of 10 times considered successful , so press the eyelid 10 times or more . In addition, there is a 5-minute interval between T ET_2-1 and T ET_2-2 on Visit2 .
* 2 : Measure only once .
*3: Use the average value of three consecutive measurements.

4.3 Bias

This is an open-label study and there are no concerns. GAT measurement is performed by the principal investigator, but at that time, the intraocular pressure of other models is not disclosed and is blinded.

4.4 Implementation of research and observation period for research subjects

period : From the date of publication of the implementation plan to December 31 , 2023

4.5 Management of research equipment

The tap-eye tonometer used in this study should be stored in a lockable space. In daily life, it cannot be assumed that the temperature and humidity of the storage environment for research equipment

will be outside the range, but avoid storage in places where sudden changes in temperature and humidity occur.

4.6 Identification of content directly entered in the case report form and interpreted as source material

Create the source materials and documents for this research accurately and at the appropriate time, and store them appropriately as verifiable records. The handling of data and the preservation of records in this research are defined as follows.

A case report form will be prepared by the principal investigator, affixed with his/her name and seal or signed, and submitted to the data management officer. The retention period for case reports shall be the day five years after the end of the study.

Observation items that serve as source materials to be described in the case report form are as follows.

-Research subject information (research subject ID, age , gender)
- Eligibility as a research subject (confirmation of inclusion and exclusion criteria)
-Intraocular pressure (3 models: TET, GAT, NCT)
-corneal thickness
-Slit lamp microscope
- Skin disorders
- Adverse events, reports from study subjects
- Presence or absence of discontinuation of case registration
- Dates and special notes for each visit

4.7Handling of personal information

Persons engaged in this research (including external parties) shall comply with the "Act on the Protection of Personal Information" and related notices that apply to the protection of personal information of research subjects. In addition, those engaged in this research must not acquire personal information by deception or other wrongful means, and will do their utmost to protect the personal information and privacy of research subjects. Obtained personal information must not be leaked without justifiable reason (the same shall apply even after the person concerned has retired from his or her position).

In addition, those engaged in this research must not handle personal information obtained in the course of conducting the research beyond the scope of consent given in advance by the research subject.

When handling personal information, the investigator must specify the purpose of its use as much as possible and keep the personal information accurate and up-to-date within the scope necessary to achieve the purpose of use. In addition, we will take necessary measures for the prevention of leakage, loss or damage of personal information and other appropriate management of personal information, and we will set out concrete implementation rules for the method of such measures.

4.8 Privacy protection of research subjects

ID number uniquely assigned by this research (research subject ID), and the personal information of research subjects will be protected. In addition, when announcing research results such as conference presentations and paper publications, we take all possible measures to protect the privacy of research subjects, such as by masking personal information in test images. Personal information about research subjects obtained in this research will not be disclosed to third parties other than those listed in the consent document when consent was obtained from the research subjects.

Five Criteria for selection/exclusion/discontinuation of research subjects

5.1 Selection criteria

Cases judged by the principal investigator to be feasible for the study.

Before entry into this study, we will be able to consent by signing the explanation consent form.

The following conditions shall be the eligibility criteria.

・Normal eyes and glaucomatous eyes

* Glaucoma study participants do not need to discontinue their daily eye drops.

・Age: 18 years old or older

・Gender: No restrictions

5.2 Exclusion Criteria

Research subjects who meet one or more of the following items will be excluded from this study:

(a) Less than 1 month after eye surgery

(b) If there is a possibility that it may be necessary to visit an ophthalmologist during the clinical research period

(c) In addition, when the principal investigator determines that the researcher is inappropriate as a subject of this research.

5.3 Termination Criteria

The criteria for discontinuing this study are set as follows.

<Cancellation of case registration>

(a) Cases in which it is determined that participation in the study is impossible by slit lamp microscopy: eg, severe cataract or glaucoma.

Lens opacity assessment used: When new opacities appear in the lens

(b) Skin evaluation: When skin disorders occur

(c) If it is determined that it is difficult to continue this study due to adverse events other than the above

(d) When the research subject requests termination or withdraws consent

(e) When the principal investigator determines that it is appropriate to discontinue this research for other reasons

No additional registrations will be made to fill the number of study subjects.

<Cancellation of research>

(a) Blindness or loss of visual function equivalent to blindness is confirmed or suspected by the tap-eye tonometer, which is the intervention device

(b) When other serious health damage is recognized or suspected

5.4 If it is unavoidable that a person who lacks the ability to give consent or a person whose voluntariness of consent is likely to be compromised becomes a subject of clinical research, clearly state the necessity of doing so.

Those who lack the ability to consent or those who are likely to lose the voluntariness of their consent are excluded from this study. In other words, the principal investigator determines that the subject is inappropriate for this study.

6 Methods of research

6.1 Methodological content of the study

This study is a non-randomised, open-label trial with test device (TET) and control device (GAT and NCT) interventions.

6.2 Combination therapy

All medical devices and drugs that have been used before participating in this study can be used together, but when measuring the tonometer of the three models (TET, GAT, NCT), remove your own glasses and contact lenses. do.

6.3 Compliance

Compliance is negligible because the use of equipment in this study is limited to the implementation facility.

7 Evaluation of efficacy

7.1 Primary endpoint

Correlation coefficient with other tonometers (coefficient of determination : R^2)

Evaluate the correlation coefficient when comparing this device with existing tonometers (GAT, NCT). The evaluation index at that time showed a statistically significant correlation ($p<0.05$) following the published paper , $R^2 \geq 0.7$ was set to be valid (Thrane et al. J Glaucoma. 2020).

7.2 Secondary endpoints

１） **Concordance rate for changes in intraocular pressure over time**

During this research period, the research subjects will be measured twice on different dates, but it is effective if the changes in intraocular pressure (Δ IOP) obtained by this device and the recognized

tonometer (GAT) match between the two devices. as an indicator of gender. The definition of agreement is based on a change of ± 2 mmHg .

An example is given. Suppose it changes from 10 mmHg to 15 mmHg in 1 month from baseline when measured with a certified product (increase of 5 mmHg). With this device, it changed from 12 mmHg to 19 mmHg (7 mmHg increase). If the definition is defined as an increase/decrease/no change with a change of ± 2 mmHg , the above example shows an increase in both models. The goal is to achieve 80% or more of the total, with the matching rate of increase/decrease/constant intraocular pressure value as an evaluation item .

２） **Repeatability in the same study subject**

Repeatability of the instrument is important for measurement reliability. At Visit 2 (1 month later), the research subject performs two measurements with an interval of 5 minutes (TET_2-1 & TET_2-2). The measurement error of TET_2-1 and TET_2-2 is evaluated by obtaining the intraclass correlation coefficient (ICC), and the target is 0.8 or more.

* In this study, corneal thickness is determined using NCT . Since corneal thickness and intraocular pressure generally show a positive correlation, evaluation of corneal thickness affects intraocular pressure.

7.3 Efficacy evaluation index evaluation, recording and analysis methods

All efficacy endpoints should be specified in the case report form. After completing the visit (baseline and one month later) during the study period, the principal investigator will contact the data management officer and request data entry and management.

8 Safety assessment

8.1 Safety evaluation index

Safety endpoints are all unfavorable medical events occurring in research subjects using this research device, regardless of whether there is a causal relationship between this research device and pharmaceuticals.

-Slit lamp microscope

- Skin disorders (periocular skin)
- Adverse events, reports from study subjects

8.2 Safety management system

During the research, the principal investigator will always keep track of the research subject's health status by providing medical care and testing on the day the research subject visits the hospital, and by securing an emergency contact method with the research subject on days other than the day the research subject visits the hospital .

If an adverse event occurs, appropriate medical care will be provided to the research subject as necessary, and the safety of the research subject will be ensured , such as by discontinuing the research subject's research.

8.3 Evaluation, recording and analysis methods for safety evaluation indicators

The principal investigator will specify and save the safety evaluation index in the case report form until the end of the observation period. Statistical analysis of data will be performed by the person in charge of statistical analysis. Qualitative parameters (slit lamp microscopy, skin disorders, adverse events) are evaluated as they are.

8.4 Diseases, etc.

Diseases, etc. refer to diseases, disabilities, deaths, or infectious diseases that are suspected to be caused by the implementation of this research, as well as abnormal laboratory test values and various symptoms. An adverse event is any unfavorable medical occurrence that occurs to a research subject when the device is used to measure intraocular pressure. It does not necessarily indicate only those that have a clear causal relationship with intraocular pressure measurement. Thus, an adverse event is any untoward or unintended sign, symptom, or illness that occurs when tonometry is performed, whether or not it is causally related to the device. If the symptoms and signs worsen after using this device, treat it as a new adverse event.

Serious adverse events refer to adverse events that fall under (1) to (5) below.

① death thing

② life threatening

By "life-threatening" is meant that the study subject was at risk of death when the event occurred.

③ Those who require hospitalization or an extension of the period of hospitalization for treatment "Hospitalization for treatment" refers to cases in which a research subject is admitted to a medical institution for one day or more due to an adverse event. On the other hand, hospitalization for examination or treatment of primary disease or complications that have not deteriorated from the state before the start of this study, hospitalization for social and convenience not for the purpose of treatment of adverse events, and before the start of this study Hospitalization for scheduled treatment or examination does not fall under "hospitalization for treatment."

④ Permanent or marked disability/malfunction

⑤ Events considered medically serious

The principal investigator reports to the accredited clinical research review board and the Minister of Health, Labor and Welfare . When reporting, take necessary measures such as explanation and treatment to research subjects.

1. Unpredictable death, disease that may lead to death, etc.
Report to: Authorized clinical research review board and Minister of Health, Labor and Welfare Report deadline: Within 7 days

2. Death, diseases that may lead to death, etc.
Report to: Accredited clinical research review committee Report deadline: Within 15 days

3. Unpredictable among the following diseases, etc.
(1) Diseases, etc. that require hospitalization at a medical institution for treatment or an extension of the period of hospitalization
(2) disability
(3) Illness, etc. that may lead to disability
(4) Death or other serious diseases, etc. corresponding to the above
(5) congenital diseases or abnormalities in subsequent generations;
Report to: Accredited clinical research review board and Minister of Health, Labor and Welfare Report deadline: Within 15 days

4. Anything other than the above
Report destination: Accredited clinical research review committee Report deadline: At the time of regular reporting

Adverse events are evaluated according to the following procedure.
Event Name: Identifies the event name of the adverse event. If a triggering disease name is specified, the event name is that diagnosis rather than the individual symptoms.
Date of onset: The date of onset shall be the date on which the adverse event occurred or was confirmed.
Severity: Evaluate the severity of adverse events in the following three grades.
1. Mild: Discomfort, but does not interfere with daily activities
2. Moderate: Discomfort to some extent that limits or affects daily life
3. Severe: Inability to work or carry out daily activities

Record whether or not any treatment (drug therapy or other treatment) was performed for the adverse event, and if so, the content of the treatment.

8.5 Observation Period for Subjects in Clinical Research after Disease Occurrence

A follow-up survey for diseases, etc. and adverse events will be conducted according to the procedures shown below.
"Recovery" means that the adverse events that occurred during the evaluation period return to their original state. To return to the state before the start.

１. If the adverse event has not resolved on the end date of the evaluation period or the date of discontinuation, the principal investigator will explain the purpose of the follow-up survey to the applicable research subject and conduct a follow-up survey after (within) 4 weeks (the implementation date is the follow-up date).
２. If the adverse event has not resolved on the day of the follow-up survey, for adverse events whose relevance to this study cannot be denied, the survey will be continued after the date of the follow-up survey until the adverse event has resolved or stabilized, if possible. If the relevance to this study can be denied, the survey will be terminated after the follow-up survey described in the preceding paragraph.
３. between the end of the observation period or the date of discontinuation and the date of follow-up survey, and the relevance to this study cannot be denied If an adverse event that cannot be denied to be related to the study becomes a “serious adverse event,” a follow-up survey will be conducted after the date of the follow-up survey until the serious adverse event resolves or stabilizes.

8.6 Reporting Bugs
55 of the Regulations for Enforcement of the Clinical Research Act , if an adverse event occurs due to a malfunction of the tap-eye tonometer used in the specified clinical research, the investigator responsible for the research will check the patient's health status and the malfunction status of the tap-eye tonometer. It must be immediately reported to the administrator of the medical institution and the manufacturing and marketing company of the product. In addition, if the defect that occurred corresponds to "disease, etc.", it shall be reported according to the procedure in " 8.4 Illness, etc.".

9 Statistical analysis

9.1 Analysis method
Correlation coefficient with other tonometers (coefficient of determination : R^2)
Evaluate the correlation coefficient (R2) when comparing this device with existing tonometers (GAT, NCT).

Concordance rate for changes in intraocular pressure over time

During this research period, the research subjects will be measured twice on different dates, but it is effective if the changes in intraocular pressure (Δ IOP) obtained by this device and the recognized tonometer (GAT) match between the two devices. as an indicator of gender. The definition of agreement is based on a change of ± 2 mmHg .

Repeatability in the same study subject

TET_2-1 and TET_2-2 is evaluated by calculating the intraclass correlation coefficient (ICC).

9.2 Number of registered cases

・ The minimum required sample size was 4 cases.

Calculated under the following conditions.

- α error (Type I) : 0.05
- β error (Type I) : 0.80
- R2 – : 0.70

9.3 Significance level

The significance level in the test for each evaluation item shall be 5% on both sides.

9.4 Study Stopping Criteria

8.4 occurs, the study will be discontinued even if the number of registered cases does not reach the planned number of cases.

9.5 Handling of test data

Visit 2 due to reasons such as discontinuation, omission or deviation , data imputation will not be performed.

Regarding the data at the time of discontinuation and dropout, the handling for tabulation and analysis is specified as follows.

- If the study was discontinued or dropped out at the prescribed visit: Aggregation and analysis as data at the prescribed visit
- If the study was discontinued or dropped out at a time other than the scheduled visit: Aggregation and analysis as data at the time of the next scheduled visit

9.6 Procedure for changing the original statistical analysis plan

・ If there are any changes from the initial statistical analysis plan, the research plan will be revised and explained in the clinical study summary report.

9.7 Analysis population

The main purpose of this study is to confirm the efficacy and safety of this device. The analysis target population in this study is defined as follows.

① Safety evaluation population

A group of cases for which the consent of the research subject has been obtained, excluding the following cases

・Patients who withdrew their consent before the baseline test

・Patients who have never used this tap-eye tonometer

② Efficacy Assessment Population (FAS)

A group of cases for which the consent of the research subject has been obtained, excluding the following cases

・Patients who withdrew their consent before the baseline test

・Patients who have never used this tap-eye tonometer

・Cases with no efficacy evaluation data

③ Efficacy Evaluation Population (PPS)
the FAS group, excluding the following cases
-Patients who discontinued participation in this study or withdrew consent after the baseline examination
・Cases in which the method and frequency of use of this tap-eye tonometer deviate from this protocol

10 monitoring

The study-related data regarding the study subject will be recorded on the case report form. The principal investigator confirms that the data are recorded accurately and affixes his/her name and seal to the case report form or signs it. Monitors must ensure that the safety and rights of research subjects are protected, that the research is conducted in compliance with the current approved research protocol and other consent forms for this research, and all applicable regulatory requirements. Make sure that

11 Ethical considerations

11.1 Advantages, Disadvantages and Side Effects of Research Subjects
<u>Benefit:</u>
The relevant research subjects of this study can share the benefit of being able to measure intraocular pressure easily and in a minimally invasive manner by using a tap-eye tonometer.

<u>Disadvantages:</u>
Possible disadvantages are described below.
・The long-term efficacy and safety of the tap-eye tonometer are unknown.

<u>Side effects:</u>
The risk of occurrence of adverse events (side effects) in this study is as follows.
- Feeling of pressure on the eyelids
・Stimulation of eyeballs

11.2 Informed Consent
Before conducting work related to this research, obtain written consent from the research subject using a consent explanation document approved by the accredited clinical research review board. If

new information that affects participation in this research is obtained after the consent of the research subject is obtained and during the period of participation in this research, the consent explanation document will be revised and the relevant information will be promptly researched. We will provide it to the subject and confirm whether or not to continue participating in this research . After obtaining the approval of the revised informed consent document from the accredited clinical research review board, the informed consent document will be used to obtain written consent from the research subject again.

If the research subject has significant visual impairment, the principal investigator will provide a written informed consent and verbally explain everything so that the research subject can understand. On top of that, if the research subject cannot fully understand, the investigator will not enroll the research subject.

11.2.1 Generic
Consent from a proxy is not required as the selection criterion is 18 years or older.

11.2.2 Informed Ascent
Not required as the selection criteria is 18 years or older.

11.3 Disclosure of research information
This research will be recorded in a database maintained by the Ministry of Health, Labor and Welfare (hereinafter referred to as " jRCT " (Japan Registry of Clinical Trials)) and published.

11.4 Responding to consultations, etc. from research subjects and their related parties
The principal investigator will respond. The direct number of the doctor's affiliation was specified in the explanatory document.

11.5 Conflicts of Interest
This research will be carried out with the funds and materials necessary for implementation from Toniji Co., Ltd., which develops and manufactures tap-eye tonometers. Details of funds, etc., are stipulated in the research consignment agreement between the implementing medical institution and Toniji Co., Ltd.
The principal investigator shall comply with the conflict of interest management regulations of the medical institution, Create a conflict of interest management plan and have it reviewed by an accredited clinical research review board. When presenting the results of this research at academic conferences or publishing papers, we will disclose the research funds and strive for transparency.

11.6 Responses to research subjects after study completion

Generally, each study subject ends the study after visit 2 . Glaucoma study subjects continue their routine practice. There is no need to discontinue eye drop therapy to participate in this study.

11.7 Dealing with Important Findings Concerning Genetic Characteristics, etc.

N/A

1 2 Data handling and record keeping

12.1 Data handling and record keeping

Create a case report form for this research accurately and at the appropriate time, and store it properly. The handling of data and the preservation of records in this research are defined as follows.

・Medical institutions can fill in the case report form prepared by the principal investigator. The principal investigator will affix his/her name and seal or sign the case report form, confirming that there is no problem with the contents of the case report form, and provide it to the data management supervisor. In addition, a copy of the case report form will be kept at the medical institution.

・The data management manager records the Excel format data input file that has been entered, corrected, and confirmed on a CD-R as the final product and reports it to the principal investigator. Specifically, two data management personnel entered the data described in the paper CRF (Case Report Form) into separate Excel files (data input files created with Microsoft Excel), and completed the input. Compare two Excel files. If the comparison does not match, enter the correct data and correct the typo (double data entry). Compare files, check, and correct input errors among business personnel. In addition, in order to prevent data falsification, etc., if there is a data correction, etc., the correction history is recorded and managed in a separate file. Discard the case report form after it has been digitized.

・The period of retention of all records by the principal investigator of the medical institution shall be 5 years after the end of this research. The case report forms to be discarded shall be shredded and discarded. Electronic data will be completely erased.

12.2 Possibility of using data for future research and providing it to other institutions

will be managed using an ID number uniquely assigned by this research (research subject ID). It is possible to use data for other research and to share data with other institutions, but the personal information of research subjects is masked data, and the principal investigator takes full responsibility.

1 3 Financial burden, insurance and other measures for research subjects

・Research subjects will not bear any financial burden when participating in this research. In addition, in order to reduce the burden on research participants due to participation in this research, we will provide a burden reduction fee (Q UO card 10,000 yen coupon).

・In the unlikely event that a health hazard occurs during the test period, appropriate treatment will be provided free of charge to the research subject. In addition, in the event of serious health damage such as death or residual disability, compensation will be paid from the clinical research insurance to which the principal investigator subscribes. ・Follow-up after the completion of this study will be switched to insurance treatment as before.

14 Arrangements for publication of information on clinical research

・All information provided or collected in this study belongs to the principal investigator.

・The investigator will disclose the summary of the summary report of this research and the summary report to jRCT , and will disclose the information by reporting the results as an academic conference presentation or a paper.

15 Addendum

No special notes.
