# Supplementary material for: Evaluation of a new transpalpebral tonometer for self-measuring intraocular pressure
Source: PLoS One. 2024 May 15;19(5):e0302568. doi: 10.1371/journal.pone.0302568 (PMC11095731; doi:10.1371/journal.pone.0302568)
Supplement: Attachment — Submitted filename: Response to reviewers_PlosOne_2023.11.2.docx [file pone.0302568.s005.docx]

Dear Academic Editor,

Dr. Georgios Labiris,

Thank you for your e-mail regarding our manuscript (PONE-D-23-21962) titled **“Evaluation of a new transpalpebral tonometer for self-measurement of intraocular pressure”** as well as for the comments from the reviewer. We believe that the paper has been much improved, largely due to the referees’ many thoughtful comments. We would like to respond below to each comment.

**To Editorial Office:**

*Journal requirements:*

*When submitting your revision, we need you to address these additional requirements.*

*1. Please ensure that your manuscript meets PLOS ONE's style requirements, including those for file naming. The PLOS ONE style templates can be found at*

*https://journals.plos.org/plosone/s/file?id=wjVg/PLOSOne_formatting_sample_main_body.pdf and*

*https://journals.plos.org/plosone/s/file?id=ba62/PLOSOne_formatting_sample_title_authors_affiliations.pdf*

Thank you.

*2. Thank you for stating the following financial disclosure:*

*“This is based on results obtained from a project, JPNP0407002, subsidized by the New Energy and Industrial Technology Development Organization (NEDO).”*

*At this time, please address the following queries:*

*a) Please clarify the sources of funding (financial or material support) for your study. List the grants or organizations that supported your study, including funding received from your institution.*

We obtained funding as JPY 24,770,681 in this grant by NEDO.

The information on NEDO is shown in the following website:

<https://www.nedo.go.jp/english/introducing/introducing_index.html>

[Page 1, lines 9-13]: One sentences has been added to the TITLE page.

“The funders had no role in study design, data collection and analysis, decision to publish, or preparation of the manuscript.”

”

*b) State what role the funders took in the study. If the funders had no role in your study, please state: “The funders had no role in study design, data collection and analysis, decision to publish, or preparation of the manuscript.”*

The statement has been added as above.

*c) If any authors received a salary from any of your funders, please state which authors and which funders.*

The statement has been added as above.

*d) If you did not receive any funding for this study, please state: “The authors received no specific funding for this work.”*

The statement has been added as above.

*Please include your amended statements within your cover letter; we will change the online submission form on your behalf.*

The statement has been added as above.

*3. Thank you for stating the following in the Competing Interests section:*

*“H.K.: CEO and equity owner, Toneasy Inc.; Patent, Toneasy Inc.”*

*Please confirm that this does not alter your adherence to all PLOS ONE policies on sharing data and materials, by including the following statement: "This does not alter our adherence to PLOS ONE policies on sharing data and materials.” (as detailed online in our guide for authors http://journals.plos.org/plosone/s/competing-interests). If there are restrictions on sharing of data and/or materials, please state these. Please note that we cannot proceed with consideration of your article until this information has been declared.*

*Please include your updated Competing Interests statement in your cover letter; we will change the online submission form on your behalf.*

The statement has been added to the revision and cover letter.

*4. We note that you have included the phrase “data not shown” in your manuscript. Unfortunately, this does not meet our data sharing requirements. PLOS does not permit references to inaccessible data. We require that authors provide all relevant data within the paper, Supporting Information files, or in an acceptable, public repository. Please add a citation to support this phrase or upload the data that corresponds with these findings to a stable repository (such as Figshare or Dryad) and provide and URLs, DOIs, or accession numbers that may be used to access these data. Or, if the data are not a core part of the research being presented in your study, we ask that you remove the phrase that refers to these data.*

Thank you for your comment. I have deleted the “data not shown” and revised the manuscript.

[Page 21, lines 361-362]: One sentence has been deleted.

~~“We are also developing an iPhone app that can communicate with the TET (data not shown).”~~

**To Reviewer #1:**

*The manuscript by Kobashi describes TapEye tonometer (TET), a novel transpalpebral self-tonometer, to obtain intraocular pressure (IOP) measurement based on palpation of the upper eyelid. They found that the corrected-IOP value measured by the TET (c-TET), calculated based on the difference between the TET and GAT measurements at the first visit, was correlated with GAT measurements. The TET has the potential to meet the need for minimally invasive IOP measurements. Although the topic area is potentially interesting, but I do have some concerns regarding the level of experimental detail provided and a lack of clarity in the presentation and description of the data that makes it difficult to establish conclusions from the study.*

Thank you for your review and positive comments. I have clarified the experimental details and modified the conclusions.

1. *Firstly, the author measure the IOPs of several 3D-printed eyeball models with various central corneal thicknesses using TET and Tono-Pen AVIA tonometers. Do these models have just eyeballs, or do they also have eyelids?*

Our 3-D printed eyeball model does not include an eyelid. To simplify the simulation of the elevated IOP model, we used artificial eye models without eyelids by changing the corneal thickness based on a 3D printing approach.

[Page 8, lines 95-97]: One sentence has been added.

“To simplify the simulation of the elevated IOP model, we used artificial eye models without eyelids by changing the corneal thickness based on a 3D printing approach.”

2. *Could the level of stiffness of the eyelids affect the measurement results and how to correct that part?*

As mentioned in the Discussion, we did not evaluate the stiffness of the eyelids, which might affect the IOP measurement obtained by the TET. To achieve accurate IOP measurement with the TET, we used the corrected-IOP value measured at the second visit as much as possible. As individual differences in thickness and stiffness of the upper eyelid occur, the TET is limited in terms of the accuracy of the measured IOP but has the advantage of easy assessment of IOP fluctuations.

[Page 22, lines 384-388]: Two sentences have been added.

“To accurately the IOP of the TET, we used the corrected-IOP value measured at the second visit as much as possible. As there are individual differences in thickness and stiffness of the upper eyelid, the TET is limited in terms of its accuracy in measuring the IOP, but is advantageous in allowing easy assessment of IOP fluctuations.”

3. *With only one glaucoma patient among the participants, how accurate is TET performance in individuals with low or high intraocular pressure? With the lowest measured value being 1.6 in the c-TET group and 10 in the GAT group, the deviation is substantial. Does this indicate inadequate accuracy of TET measurements in cases of low intraocular pressure?*

Thank you for your advice. As you mentioned, the TET measurements did not indicate the suitable accuracy in eyes with lower IOP of the present prototype. Indeed, the example with the lowest measured c-TET value of 1.6 is described as follows. A 75 years old male without glaucoma had an IOP of 10 and 18.01 mmHg with the GAT and TET at visit 1, respectively (ΔIOP=8.01). At visit 2, his IOP measured 10 and 9.62 mmHg with the GAT and TET, respectively. Thus, c-TET was 1.61 mmHg. The reason for the difference in IOP might be due to the patient learning how to operate the device at visit 1 and his sagging eyelid. Since we need to be careful about ΔIOP in eyes with lower IOPs, ΔIOP might induce large errors in c-TET at visit 2.

[Page 20, lines 321-328]: Seven sentences have been added.

“In eyes with lower IOP, TET measurements did not indicate adequate accuracy of the present prototype. Indeed, the example with the lowest measured c-TET value of 1.6 is described as follows. A 75 years old male without glaucoma had an IOP of 10 and 18.01 mmHg with the GAT and TET at visit 1, respectively (ΔIOP=8.01). At visit 2, his IOP measured 10 and 9.62 mmHg with the GAT and TET, respectively. Thus, c-TET was 1.61 mmHg. The reason for the difference in IOP might be due to the patient learning how to operate the device at visit 1 and his sagging eyelid. Since we need to be careful about ΔIOP in eyes with lower IOPs, ΔIOP might induce large errors in c-TET at visit 2.”

4. *The subjects can seek help or receive instructions from technicians when using TET in both visits. How do they perform if there is no access to TET instructions? Are the results still reliable?*

When the subjects asked for help or held the device in an inappropriate manner and pushed the button, the technicians gave the proper instructions while standing by their side at both visits. The reliability of the TET depended on the user skill in the present prototype, as the repeatability showed moderate to good results.

[Page 11, lines 162-164]: One sentence has been modified.

“When the subjects asked for help or held the device in an inappropriate manner and pushed the button, the technicians gave the proper instructions while standing by their side at both visits.”

[Page 22, lines 378-389]: One sentence has been added.

“The reliability of TET depended on the user skill with the present prototype, as the repeatability showed moderate to good results.”

5. *How many times of measurements are needed to obtain a TET IOP value which successfully pass the criteria? In a single measurement session, what range of differences between the data points is considered acceptable? Do the failure measurements affect the IOP value before a successful TET measurement is obtained?*

At least seven successful measurements were required to obtain the IOP value with the TET. The average value of seven measurements was considered the IOP value for the TET. Failed measurements were excluded by the IOP calculation.

[Page 10, lines 149-151]: Two sentences have been added.

“The average value of the seven measurements was considered the IOP value of the TET. Failed measurements were excluded by the IOP calculation.”

6. *The significant correlation between TET and GAT seems to rely on correction based on the difference between the TET and GAT measurements in visit 1. In this study, the visit interval is one month. Is the correlation between c-TET and GAT still significant if the visit interval is prolonged?*

It is not clear whether the significant correlation between c-TET and GAT would be maintained for visit intervals longer than 1 month. To reduce the difference between the TET and GAT measurements, we focused on the ΔIOP at visit 1 and optimized c-TET at visit 2.

[Page 19, lines 305-307]: One sentence has been added.

“It is not clear whether the significant correlation between c-TET and GAT would be maintained for visit intervals longer than 1 month.”

7. *It is mentioned that no vision-threatening complications were observed in this study. What is the incidence of abrasions, eyelid disorder, and conjunctivitis in this study?*

Other adverse events, such as abrasions, eyelid disorder, and conjunctivitis, did not occur in any subject at visits 1 and 2.

[Page 19, lines 292-293]: One sentence has been added.

“Other adverse events such as abrasions, eyelid disorder, and conjunctivitis did not occur in any subject at visit 1 and 2.”

8. *Patients with a history of incisional glaucoma surgery, LASIK, corneal surgery, or history of previously diagnosed keratoconus were not excluded in this study. How does the TET perform when measuring these patients? Is the GAT sufficiently accurate as gold standard considering these patients?*

As you mentioned, patients with a history of incisional glaucoma surgery, LASIK, or corneal surgery were not excluded, nor were patients who had undergone cataract extraction at least one month prior. We did not exclude patients with a history of previously diagnosed keratoconus. However, there were only 2 patients with nonprogressive keratoconus, 2 patients with LASIK, and 1 patient with normal-tension glaucoma treated with eye drops. We could not compare the IOPs obtained with the TET of these patients with those of normal subjects because of the small sample size. Generally, compared with those of the GAT, corneal biomechanics measurements are recommended in patients with a history of corneal surgery and keratoconus in terms of the evaluation of IOP.

[Page 20, lines 328-334]: Three sentences have been added.

“Only 2 patients in our group had nonprogressive keratoconus, 2 patients had undergone LASIK, and 1 patient had normal-tension glaucoma treated with eye drops. We could not compare the IOPs obtained with TET for these patients with those obtained from normal subjects because of the small sample sizes. Generally, compared with GAT, corneal biomechanics measurements are recommended in patients with a history of corneal surgery and keratoconus in terms of the evaluation of the IOP.”

9. *In line 287, the author claimed that our TET can detect the dynamic change in IOP in patients with glaucoma or ocular hypertension. However, the participants’ IOP measured by GAT all falls within the range of 22 or below. Insufficient information is available to draw the conclusion mentioned above.*

This sentence has been deleted because of its exaggerated tone. Our goal is to detect the dynamic change in IOP in patients with glaucoma or ocular hypertension in future work.

[Page 19, lines 312-314]: One sentence has been added.

“Our goal is to detect the dynamic changes in the IOP in patients with glaucoma or ocular hypertension in future work.”

[Page 19, lines 310-312]: One sentence has been deleted.

~~“Similar to the use of an individually owned blood pressure monitor in daily life, our TET can detect the dynamic change in IOP in patients with glaucoma or ocular hypertension.”~~

10. *Of eyelid tonometer TGDc-01 IOP values, 66.4% (132 eyes) within ±2 mmHg and 81.0% (161 eyes) within ±3 mmHg of those with applanation tonometry, and 10.0% (20 eyes) the deviation from applanation tonometry was more than 4 mmHg. Only 50.5% of c-TET measurements were within 2.5 mmHg of GAT measurements. What are the pros and cons of TET compared with TGDc-01?*

We cannot speak of the superiority of the measurement accuracy between TET and TGDc-01 because the data between the two have yet to be compared. The TGDc-01 is not useful as an instrument for self-measurement, as an examiner needs to place the tip of the instrument on the patient’s upper lid margin. Compared with the TGDc-01, the TET has the advantage of self-measurement safely after sufficient training and practice.

[Page 21, lines 343-348]: Three sentences have been added.

“The superiority in terms of the measurement accuracy between the TET and TGDc-01 is unknown since, to date, the data have yet to be compared. The TGDc-01 is not useful as an instrument for self-measurement by patients, as the examiner places the tip of the instrument on the patient’s upper lid margin. Compared with the TGDc-01, the TET has the advantage of allowing self-measurement safely after sufficient training and practice.”

11. *What are the factors related to* *difference between the TET and GAT measurements? Do eyelid thickness or central corneal thickness affect difference between the TET and GAT measurements?*

No significant factors were detected when we looked for an association with ΔIOP (TET-GAT) in the present study. The central corneal thickness was not significantly associated with ΔIOP. Unfortunately, we did not evaluate the eyelid thickness in each subject. As individual differences in thickness and stiffness of the upper eyelid occur, TET has a limitation in terms of accuracy of IOP.

[Page 22, lines 386-388]: One sentence has been added.

“As there are individual differences in thickness and stiffness of the upper eyelid, the TET is limited in terms of the accuracy of the measured IOP but has the advantage of easy assessment of IOP fluctuations.”

12. *In figure 6, the label corresponding to the left fourth column may be incorrect.*

Thank you. We have modified Figure 6.

*13. As Kobashi designed the research, collected data, and also analysis and interpretation, How could you reduce bias and improve confidence?*

To reduce bias and improve confidence, we propose that independent researchers conduct similar clinical studies and evaluate the efficacy and usability of the TET. When we move to the next phase, some independent researchers should demonstrate the clinical advantages of the TET.

[Page 23, lines 392-397]: Three sentences have been added.

“Fourth, as an inventor of the TET, Kobashi designed the research, collected the data, and also performed analyses and interpretations in the present study. In order to reduce bias and improve confidence, we suggest that independent researchers conduct similar clinical studies and evaluate the efficacy and usability of the TET. When we move to the next phase, some of the independent researchers in our research team should demonstrate the clinical evidence of the advantages of the TET.”

**To Reviewer #2:**

*It tis a interesting device for intraocular pressure measurement. There are some opinios：*

Thank you for your review and positive comments. I have clarified the experimental details and modified the conclusions.

1） *When making aTET or GAT measurement, whether it is measured once or three times to take the average value?*

At least seven successful measurements were required to obtain the IOP value with the TET. The average value of the seven measurements was considered the IOP value of the TET. Failed measurements were excluded by the IOP calculation. With the GAT, only a single measurement was performed at each visit.

[Page 10, lines 149-151]: Two sentences have been added.

“The average value of the seven measurements was considered the IOP value of the TET. Failed measurements were excluded by the IOP calculation.”

[Page 12, lines 173-174]: One sentence has been added.

“With the GAT, only a single measurement was performed at each visit.”

2） *How to consider the factors of the eyelid, whether the single eyelid or double eyelid is considered when selecting a patient?*

At this development stage, we do not consider factors such as single or double eyelid. We assume that the TET measurements may be affected by the thickness and stiffness of the eyelids, which vary among individuals; this effect would pertain specifically to the transpalpebral approach.

*3）The abscissa in Figure 7 is mean of c-TET and GAT, but in Figure 5 it is GAT. This is unreasonable. If Fig7 is a Bland-Altmen plot to illustrate consistency, you can provide an additional correlation plot with GAT as abscissa.*

Thank you for the important advice. In Fig 7, we have added the additional correlation plots between the values of (c-TET-GAT) and GAT at visit 2. As shown in these plots, no statistical correlation was found between these two parameters.

[Page 18, lines 272-274]: One sentence has been added.

“However, no statistical correlation was found between the values of (c-TET-GAT) and GAT at visit 2 in both the right and left eyes (**Figure 7 C, D**).”

[Page 18, lines 274-277]: One sentence has been deleted.

“~~Compared with the GAT measurements, the c-TET measurements showed a weak but significant tendency to overestimate the IOP in eyes with higher IOP values (>17 mmHg) and underestimate the IOP in those with lower IOP values (<17 mmHg).”~~

[Page 21-22, lines 363-367]: Two sentences have been added.

“Since there were no statistical correlations between the values of (c-TET-GAT) and GAT at visit 2, our Bland‒Altman analysis did not demonstrate consistency (Figure 7). As for Icare, the measurements obtained with the rebound tonometer underestimated the IOP with respect to the GAT reading, whereas the measurement showed an increasing overestimation of the IOP in comparison to the GAT measurement.^16^”

[Page 22, lines 368-374]: Two sentences have been deleted.

~~“In the Bland‒Altman analysis, the c-TET showed a general tendency to overestimate IOPs when the GAT reading was in the range of 0.4 to 0.5 mmHg. In lower to moderate IOP ranges (<17 mmHg), c-TET measurements underestimated IOP in relation to the GAT reading, whereas the TET measurement showed an increasing overestimation of IOP in comparison to the GAT measurement for IOPs >17 mmHg (Figure 7). Similar results were shown with the rebound tonometer.~~^~~16~~^ ~~The TET needs to evaluate larger data and optimize the IOP by calculating the differences.”~~

[Page 27, lines 494-497]: One sentence has been added to the figure legends.

“Correlation plot: C, Scatterplot between the values of (c-TET-GAT) and GAT at visit 2 in the right eye (Spearman r=-0.135, P=0.178). D, Scatterplot between the values of (c-TET-GAT) and GAT at visit 2 in the left eye (Spearman r=-0.192, P=0.054).”

*4）In Figure 7，although most of the points fall between 95% limits of agreement, the deviation from the upper and lower limits actually far exceeds the clinical approval value*

When we looked at the accuracy of IOP, the agreement was not adequate for clinical use given how much the deviation from the upper and lower limits exceeded the clinical approval value. However, our goal is to detect the dynamic change in IOP in patients with glaucoma or ocular hypertension in future work. If this becomes possible, the TET will have certain competitive advantages in terms of its ability to track relative changes in IOP and its safety of use. We are conducting a new study following the changes in IOP before and after glaucoma drops using the TET and GAT.

[Page 19, lines 312-314]: One sentence has been added.

“Our goal is to detect dynamic changes in IOP in patients with glaucoma or ocular hypertension in future work.”

*5）In the study, the NCT test method was also used. It is recommended to provide the consistency analysis results of NCT and GAT as a reference to clarify the equipment needs to be improved.*

To confirm the consistency in the IOP obtained between the NCT and GAT, we statistical correlation analysis. There were significant correlations between the IOPs obtained with the NCT and GAT at each visit in both right and left eyes (**please see Table 3**).

**Table 3.** Correlation of IOPs obtained from the noncontact tonometer and Goldmann applanation tonometer.

| **Visit** | **Spearman correlation coefficient** | **P value** |
| --- | --- | --- |
| Right eye at visit 1 (n=104) | 0.521 | <0.001 |
| Left eye at visit 1 (n=104) | 0.427 | <0.001 |
| Right eye at visit 2 (n=101) | 0.446 | <0.001 |
| Left eye at visit 2 (n=101) | 0.530 | <0.001 |

IOP=intraocular pressure.

[Page 13, lines 196-197]: One sentence has been added.

“To confirm the consistency in the IOP between the NCT and GAT, we performed statistical correlation analysis.”

[Page 17, lines 257-258]: One sentence has been added.

“The IOPs between the NCT and GAT were significantly correlated at each visit in both the right and left eyes (**Table 3**).”

We believe the manuscript has been prepared and submitted satisfactorily and hope that it will be accepted for publication in *Plos One*. Thank you for your attention and consideration.

Sincerely yours,

Hidenaga Kobashi, MD, PhD, Department of Ophthalmology, Keio University, School of Medicine, Tokyo, Japan. E-mail address: hidenaga_kobashi@keio.jp
