# Supplementary material for: Evaluation of a new transpalpebral tonometer for self-measuring intraocular pressure
Source: PLoS One. 2024 May 15;19(5):e0302568. doi: 10.1371/journal.pone.0302568 (PMC11095731; doi:10.1371/journal.pone.0302568)
Supplement: Attachment — Submitted filename: Response to reviewers_PlosOne_2024.3.1.docx [file pone.0302568.s006.docx]

Dear Academic Editor,

Dr. Georgios Labiris,

Thank you for your e-mail regarding our manuscript (PONE-D-23-21962R1), titled **“Evaluation of a new transpalpebral tonometer for self-measuring intraocular pressure”,** as well as for the comments from the reviewers. We believe that the paper has been greatly improved, largely due to the referees’ many thoughtful comments. We would like to respond below to each comment.

**To Reviewer #3:**

*Thank you for the opportunity to provide a review of Manuscript PONE-D-23-21962 entitled "Evaluation of a new transpalpebral tonometer for self-measurement of intraocular pressure." My comments relate primarily to the adequacy of the implementation and reporting of epidemiologic and statistical procedures.*

Thank you for your review and positive comments. I have clarified the experimental representations in this revision.

*I understand that this manuscript underwent a previous round of peer review. I have reviewed both the original and revised versions of the manuscript, including the author's response to the first-round comments.*

Modifications have been made based on the comments of all the reviewers.

*The quality of the technical English is appropriate but variable. While offering no bar to my evaluation of the manuscript, many errors were present. I strongly recommend that the authors conduct a thorough round of copyediting to eliminate the grammatical and syntactical errors in the text.*

To eliminate grammatical and syntactical errors in the text, this revision was examined by a peer review of native English experts.

***# Reporting of correlation results***

*The authors state that they will report Pearson's correlation coefficient (or Spearman's). However, the authors do not report the coefficient of correlation (r) in any of the scatterplots provided, but instead report the coefficient of determination (r^2). (See Figure 4, all graphs in Figure 5, and all graphs in Figure 6.)*

*The authors need to clarify whether this is a labelling or calculation error. That is, did they actually calculate r^2 or did they simply label the r results as r^2. It is imperative that they DO NOT present r^2 results because the meaning of the coefficient of determination is very different from the coefficient of correlation. This is a major error that requires careful attention.*

I apologize for the confusion. The coefficient of determination (R^2^) was deleted in each figure. Spearman's correlation coefficients are now reported in the figure legends.

***# Description of the device and the process of use***

*Still images are provided by the authors to describe the device and demonstrate its usage. This is substandard. The journal allows for the submission of video clips, and this is one time when such a submission is preferable to the textual and still image descriptions of the device and its use.*

I have added a video illustrating the device and the process of its use as a supplemental video.

***# Consistency of presentation of means and standard deviations***

*The authors state in the methods that they will present outcomes as means plus-minus standard deviations. The authors inconsistently carry this approach throughout the text. In many places, the authors report the mean (SD). The authors need to observe consistency in their reporting.*

The outcomes are presented as the means ± standard deviations in this revision.

*# Recommendation*

*The issues identified above are relatively manageable, but I cannot support the acceptance of the manuscript until they are addressed.*

The above issues have been addressed.

We believe that the manuscript has been satisfactorily prepared and submitted, and we hope that it will be accepted for publication in *PLoS ONE*. Thank you for your attention and consideration.

Sincerely yours,

Hidenaga Kobashi, MD, PhD, Department of Ophthalmology, Keio University, School of Medicine, Tokyo, Japan. E-mail address: hidenaga_kobashi@keio.jp
